# The plastidial pentose phosphate pathway is essential for postglobular embryo development in *Arabidopsis*

**DOI:** 10.1073/pnas.1908556116

**Published:** 2019-07-11

**Authors:** Vasilios M. E. Andriotis, Alison M. Smith

**Affiliations:** ^a^John Innes Centre, NR4 7UH Norwich, United Kingdom;; ^b^School of Natural and Environmental Sciences, Newcastle University, NE1 7RU Newcastle-upon-Tyne, United Kingdom

**Keywords:** *Arabidopsis*, embryo, nucleotide synthesis, plastid, pentose phosphate pathway

## Abstract

Large numbers of genes essential for embryogenesis in *Arabidopsis* encode enzymes of plastidial metabolism. Disruption of many of these genes results in embryo arrest at the globular stage of development. However, the cause of lethality is obscure. We examined the role of the plastidial oxidative pentose phosphate pathway (OPPP) in embryo development. In nonphotosynthetic plastids the OPPP produces reductant and metabolic intermediates for central biosynthetic processes. Embryos with defects in various steps in the oxidative part of the OPPP had cell division defects and arrested at the globular stage, revealing an absolute requirement for the production via these steps of ribulose-5-phosphate. In the nonoxidative part of the OPPP, ribulose-5-phosphate is converted to ribose-5-phosphate (R5P)—required for purine nucleotide and histidine synthesis—and subsequently to erythrose-4-phosphate, which is required for synthesis of aromatic amino acids. We show that embryo development through the globular stage specifically requires synthesis of R5P rather than erythrose-4-phosphate. Either a failure to convert ribulose-5-phosphate to R5P or a block in purine nucleotide biosynthesis beyond R5P perturbs normal patterning of the embryo, disrupts endosperm development, and causes early developmental arrest. We suggest that seed abortion in mutants unable to synthesize R5P via the oxidative part of the OPPP stems from a lack of substrate for synthesis of purine nucleotides, and hence nucleic acids. Our results show that the plastidial OPPP is essential for normal developmental progression as well as for growth in the embryo.

## Significance

Many mutations that affect plastidial metabolism are embryo-lethal, as expected if the disrupted genes encode enzymes with essential housekeeping functions. However, some mutations that disrupt the plastidial oxidative pentose phosphate pathway (OPPP) cause developmental defects, as well as embryo arrest at the globular stage of development. We show that the OPPP provides the substrate for the pathway of purine synthesis, ribose-5-phosphate, and is thus essential for the generation of nucleic acids during the very early stages of embryo development. Inadequate purine synthesis leads to abnormal patterns of cell division in the embryo and blocks development beyond the globular stage. Therefore, defects in primary metabolic pathways can have profound consequences for development as well as simply reducing growth.

**Fig. 1.**
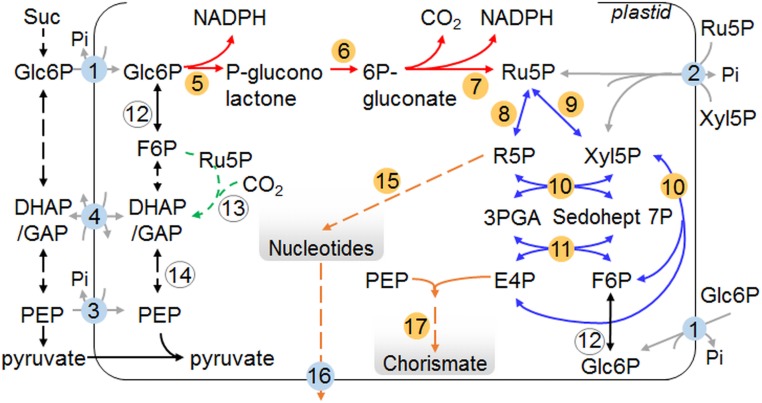
Overview of the plastidial OPPP and related pathways. Dashed lines indicate multiple enzymatic steps that for simplicity are omitted. Arrows: red and blue, oxidative and nonoxidative sections of the OPPP, respectively; black, glycolysis and related reactions; green, partial Calvin/Benson cycle: in heterotrophic plastids Rubisco catalyses the conversion of CO_2_ lost in respiration to 3PGA, which can enter plastidial glycolysis (61); gray, plastid envelope transporters; orange, pathways for nucleotide and chorismate synthesis. Numbers: yellow, enzymes discussed in text; blue, transporters at the plastid envelope. 1: GPT1, Glc6P transporter; 2: XPT, pentose phosphate transporter; 3: PPT, phosphoenolpyruvate transporter; 4: TPT, triose phosphate transporter; 5: G6PDH, Glc6P dehydrogenase; 6: PGL3, 6-phosphogluconolactonase; 7: PGD, 6-phosphogluconate dehydrogenase; 8: RPI, ribose 5P isomerase; 9: RPE3, ribulose 5P epimerase; 10: TKL, transketolase; 11: transaldolase; 12: PGI, phosphoglucose isomerase; 13: Rubisco; 14: phosphoglyceromutase and enolase; 15: PUR5, 5-aminoimidazole ribonucleotide (AIR) synthase; 16: BT1, plastidial adenine nucleotide transporter; 17: CS, chorismate synthase.

Large numbers of genes (called *EMB* genes) are essential for embryo development in *Arabidopsis*: *emb* mutants undergo developmental arrest before maturity, leading to seed abortion, nonviable seeds, or nonviable seedlings (1–6). Many *EMB* genes encode proteins directly involved in fundamental growth and development processes, including transcription, nucleic acid synthesis and replication, protein translation and transport, cellular differentiation, and organ morphogenesis, but others encode enzymes of primary carbohydrate metabolism, notably the plastidial oxidative pentose phosphate pathway (OPPP) (Fig. 1). The embryo lethality of mutants defective in plastidial OPPP components is surprising since OPPP enzymes are encoded by multiple genes and OPPP substrates and products can be derived by more than 1 metabolic route. Because of this high level of redundancy, loss of individual components might be expected to have minimal phenotypic consequences. Nonetheless, mutants lacking plastidial isoforms of 6-phosphogluconolactonase (PGL3) and ribose-5-phosphate (R5P) isomerase (RPI3) are listed in the SeedGenes collection of embryo-lethal mutants (http://seedgenes.org/) (3, 4, 7, 8), and we showed that embryos arrest early in development in response to partial loss of the plastid envelope glucose-6-phosphate (Glc6P) transporter (GPT1) that provides the substrate for the OPPP (9). These observations imply that the first oxidative part of the plastidial OPPP (conversion of Glc6P to ribulose 5-phosphate [Ru5P]) (Fig. 1) has a specific, essential, but unknown role during embryogenesis.

One possible reason for the requirement for the oxidative part of the plastidial OPPP during embryo development is that it provides substrates for essential biosynthetic pathways that are wholly or partly plastidial. Intermediates of the nonoxidative part of the OPPP are substrates for the biosynthesis of nucleotides, histidine, aromatic amino acids and related compounds, some vitamins, and hormones. Mutations directly affecting these pathways often are embryo-lethal (4, 10, 11). However, substrates for these pathways can be generated in the plastid by routes that do not involve the oxidative part of the OPPP. In addition to Glc6P, the plastid has the capacity to import 3-phosphoglycerate (3PGA) and triose phosphates, phosphoenolpyruvate, pyruvate, and the 5-carbon phosphorylated intermediates, Ru5P and xylulose 5-phosphate (Xyl5P) (12–17). Together, these compounds potentially allow generation inside the plastid of all of the intermediates of the nonoxidative part of the OPPP independently of the metabolism of imported Glc6P via the oxidative part of the OPPP (Fig. 1).

A second role that could explain the requirement for the oxidative part of the OPPP is the provision of reducing power inside the plastid. Conversion of Glc6P to Ru5P generates NADPH necessary for plastidial biosynthetic pathways, including the synthesis of vitamin K1, tocopherols, fatty acids, and aromatic amino acids. However, reductant could also or alternatively be imported from the cytosol through redox shuttles (18, 19). A major demand for reductant is also imposed by chlorophyll synthesis, which occurs as embryo cell division ceases and differentiation starts. In the final stages of chlorophyll synthesis and thylakoid assembly, protochlorophyllide is converted to chlorophyllide by protochlorophyllide oxidoreductase (20). Protochlorophyllide is highly unstable, and decays with the release of singlet oxygen (reactive oxygen species, ROS) in the presence of light. It is believed that damaging ROS production (which can alter developmental patterns and trigger programmed cell death, PCD) (21–23) is prevented by 2 NADPH-dependent processes: stabilization of protochlorophyllide by complex formation with protochlorophyllide oxidoreductase, and rapid conversion of protochlorophyllide to chlorophyllide (24, 25). Taken together with our observation that PCD-like growth arrest coincides with chlorophyll accumulation in embryos with partial GPT1 loss (9), these considerations led us to suggest previously that the essential role of the oxidative part of the plastidial OPPP might be NADPH production to prevent ROS damage during chlorophyll synthesis.

The aim of this work was to test the above suggestions about why the plastidial OPPP is so important for embryo development. To this end, we assembled a collection of *emb* mutants lacking either plastidial OPPP proteins or proteins of putatively downstream biosynthetic pathways. We also analyzed mutants lacking enzymes and transporters that might provide substrates for these biosynthetic pathways via alternative routes. All mutants were grown in the same conditions to ensure that distinct terminal phenotypes resulted from genetic and developmental rather than environmental differences. From systematic characterization of the timing and nature of embryo arrest in these mutants, we conclude that the essential function of the plastidial OPPP is the provision of substrates for purine nucleotide biosynthesis in early embryo development, without which developmental progression is blocked before morphogenesis is complete. Flux through alternative routes of substrate provision is unable to meet the high demand for nucleotides during the cell-division phase of seed development.

## Results

### Embryos Lacking Plastidial PGL3 Abort at the Globular Stage.

**Fig. 2.**
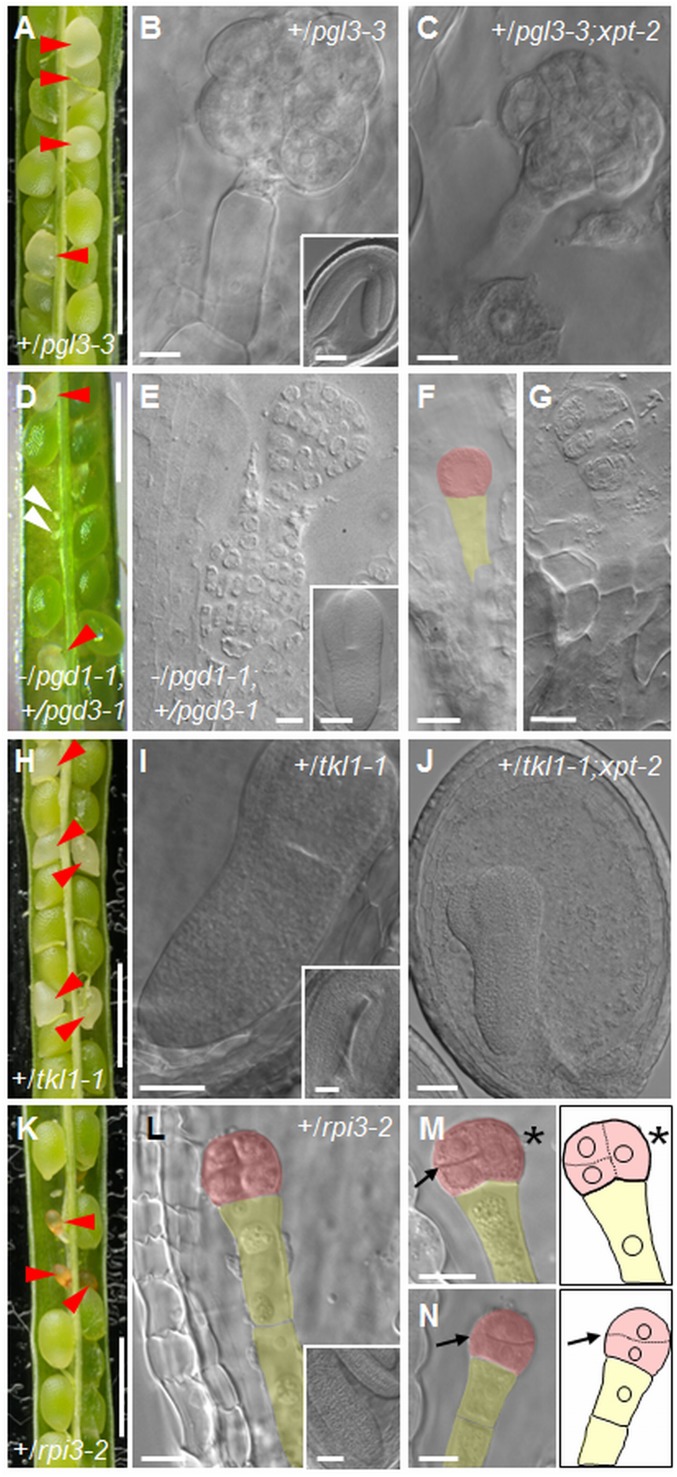
Phenotype of seeds in siliques on heterozygous plants. (*A*) Silique from a +/*pgl3-3* plant. (*B*) Raspberry-like embryo from a white seed from *A* (*Inset*: embryo from a green seed from *A*) and (*C*) from a white seed from a silique on a +/*pgl3-3;xpt-2* plant. (*D*) Silique from a *−/pgd1-1;+/pgd3-1* plant. (*E*) Raspberry-like embryo from a white seed in a silique from a *−/pgd1-1;+/pgd3-1* plant (*Inset*: embryo at the torpedo stage from a green seed from *D*). (*F*) One-cell stage embryo and (*G*) octant stage embryo from aborted seeds in a silique from a *−/pgd1-1;+/pgd3-1* plant. (*H*) Silique from a +/*tkl1-1* plant. (*I*) Aborting torpedo-stage embryo from a white seed in *H* (*Inset*: embryo from a green seed from *H*) and (*J*) from a silique on a +/*tkl1-1;xpt-2* plant. (*K*) Silique from a +/*rpi3-2* plant. (*L*) Octant-stage embryo from an aborting seed in *K* (*Inset*: embryo from a green seed from *K*). (*M*) Embryo from an aborting seed as in *K*, in which the first division in the embryo proper was longitudinal (asterisk) and the second transverse (arrow). A drawing is included for clarity. (*N*) As in *M*, but for an embryo with transverse rather than longitudinal division planes (arrow). Red arrowheads in *A*, *D*, *H*, and *K*: abnormal or aborting seeds; open arrowheads in *D*: unfertilized or aborted ovules. Embryos in *B*, *C*, *E*, *G*, *I*, *J*, and *L*–*N* were imaged with DIC optics. False color in *F* and *L*–*N* highlights the embryo proper (red) and the suspensor (yellow). (Scale bars: *A*, *D*, *H*, and *K* are 1 mm; *E* is 7.5 μm; *B*, *C*, *G*, *F*, and *L*–*N* are 10 μm; *I* and *J* are 25 μm; and *Insets* in *B*, *I*, and *L* are 50 μm.)

To discover the importance of the oxidative part of the plastidial OPPP for development through the globular stage, we examined embryos in mutants lacking plastidial PGL (step 6 in Fig. 1) (26). One of the 5 *PGL* genes in *Arabidopsis*, *PGL3* (At5g24400), encodes an enzyme with dual plastidial and peroxisomal localization (8, 27, 28), whereas the other 4 encode cytosolic proteins. Mutations eliminating PGL3 are embryo-lethal due to loss of the plastidial but not the peroxisomal activity (8). We found that developing siliques from plants heterozygous for 2 independent mutations in *PGL3*, +/*pgl3-2* and +/*pgl3-3* (*SI Appendix*, Fig. S1) (8), contained about 25% white seeds, which collapsed during maturation (Fig. 2*A*, Table 1, and *SI Appendix*, Fig. S2*B* and Table S1). Embryos from white seeds progressed only to the globular stage. Similar to embryos with reduced GPT1 activity (9), aborting *pgl3* embryos lacked normal embryonic tissues and had a raspberry-like appearance due to irregularly shaped cells in the outer cell layer of the embryo proper (protoderm) (Fig. 2*B* and *SI Appendix*, Fig. S2*D*). These defects were first seen at 4 d after flowering (DAF), when protoderm cells became abnormally enlarged (*SI Appendix*, Fig. S3). At this point, almost 80% of wild-type (Col-0) embryos but only 60% of embryos from *+/pgl3-3* plants had progressed to the transition-heart stage (*SI Appendix*, Figs. S3 and S4 *A* and *B*). Beyond 4 DAF, embryos in phenotypically normal seeds of *+/pgl3-3* progressed to maturity at the same rate as embryos from wild-type plants, whereas embryos in white seeds remained at the globular stage (*SI Appendix*, Figs. S3 *A*–*D* and S4 *A* and *B*, and Table S4). In contrast with +/*pgl3-2* and +/*pgl3-3*, all seeds were phenotypically normal on homozygous plants carrying a third mutant allele of *PGL3*, *pgl3-1*, which strongly reduces but does not eliminate plastidial PGL activity (*SI Appendix*, Figs. S1 and S4*C*) (28). Hence, Glc6P metabolism via the oxidative part of the plastidial OPPP as far as 6-phosphogluconate is essential for normal development through the globular stage.

**Table 1.** Summary of mutant lines used in this study

AGI code	Gene	Protein/enzyme	Step*	Mutant lines		Eco-type	Position^†^
At5g17630	*XPT*	Xul5P/phosphate translocator	2	*xpt-2*	SAIL_378_C01	Col	+68
				*xpt-4*	GT_5_112515	Ler	+1017
At5g24400	*PGL3*	6-Phosphogluconolactonase	6	*pgl3-1*	SALK_005685	Col	+975
				*pgl3-2*	FLAG_219G10	WS	+250
				*pgl3-3*	emb2024^‡^	Col	+215/437^§^
At1g64190	*PGD1*	6-Phosphogluconate dehydrogenase	7	*pgd1-1*	GABI_762C02	Col	+11
				*pgd1-2*	SALK_121521	Col	+1445
				NA^¶^	SALK_002712	Col	−107
At5g41670	*PGD3*	6-Phosphogluconate dehydrogenase	7	*pgd3-1*	SAIL_528_E08	Col	+20
				*pgd3-2*	SALK_040050	Col	+309
				*pgd3-3*	SALK_202519	Col	+334
At3g04790	*RPI3*	d-Ribose-5-phosphate isomerase	8	*rpi3-1*	emb3119-1^‡^	Nössen	+631
				*rpi3-2*	emb3119-2^‡^	Col	+4
At5g61410	*RPE3*	d-Ribose-5-phosphate epimerase	9	*rpe3-2*	emb2728-2^‡^	Col	+146
				*rpe3-3*	SAIL_240_G08	Col	+147^#^
At3g60750	*TKL1*	Transketolase	10	*tkl1-1*	WiscDsLox453_456I14	Col	+2750
				*tkl1-2*	SAIL_58_D02	Col	+509
At3g55010	*PUR5*	AIR synthase	15	*pur5-1*	SALK_070673	Col	+855
				*pur5-2*	SAIL_343_A07	Col	+1498
At4g32400	*BT1*	Brittle1	16	*bt1*	SALK_078655	Col	+1069
At1g48850	*CS*	Chorismate synthase	17	*cs-1*	emb1144^‡^	Col	
At3g54470	*UMPS*	Orotate phosphoribosyltransferase	18	*umps-1*	FLAG_038G05	WS	+1808

**Fig. 3.**
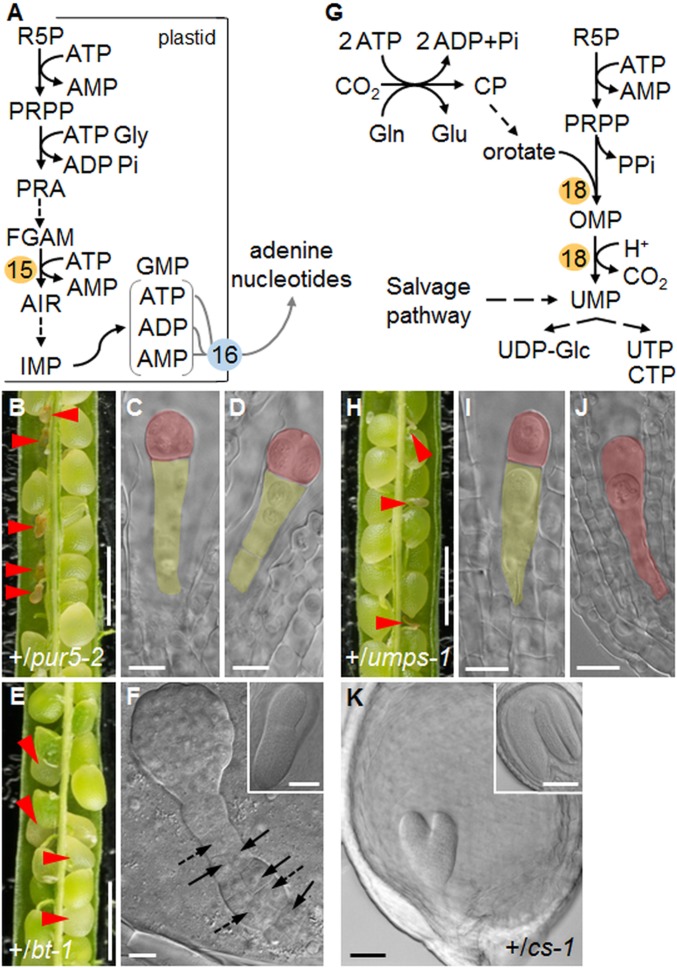
Nucleotide synthesis and embryo development. (*A*) Overview of purine biosynthesis. PPRP: 5-phosphoribosyl-1-pyrophosphate; PRA: 5-phosphoribosylamine; FGAM: formylglycinamidine ribotide; AIR: 5-aminoimidazole ribotide. 15: PUR5, AIR synthase. 16: BT1. (*B*) Silique from a +/*pur5-2* plant. (*C* and *D*) Embryos from aborting seeds in *B* at the 1-cell (*C*) or 2-/4-cell (*D*) stage. Siblings from green seeds in the same silique were at the upturned-U stage. (*E*) Silique from a +/*bt-1* plant. (*F*) Raspberry-like embryo from a white seed in *E*. Arrows and dashed arrows: abnormal longitudinal and oblique cell division planes, respectively, in the suspensor. (*Inset*) Torpedo-stage embryo from a green seed from *E*. (*G*) Overview of pyrimidine synthesis. For simplicity, plastidial, mitochondrial, and cytosolic compartmentation of individual reactions is not shown. CP: carbamyl phosphate, OMP: orotidine 5-monophosphate. 18: UMPSase. (*H*) Silique from a +/*umps-1* plant. (*I*) Embryo at the 1-cell stage and (*J*) zygote from aborted seeds in *H*. Embryos from green seeds were at the torpedo stage. (*K*) Embryo from a pale green seed in a silique from a +/*cs-1* plant (*Inset*: upturned-U stage embryo from a normal seed). Red arrowheads: abnormal or aborting seeds. Embryos in *C*, *D*, *F*, *I*, *J*, and *K* were imaged with DIC optics. False color highlights the embryo (red) and the suspensor (yellow). (Scale bars: *B*, *E*, and *H* are 1 mm; *C*, *D*, *F*, *I*, and *J* are 10 μm; and *K* and *Insets* in *F* and *K* are 50 μm.)

* Steps refer to the numbered reactions shown in Figs. 1 and 3.

^†^ Position refers to the number of nucleotides relative the ATG start codon at which the T-DNA was confirmed to be inserted by sequencing flanking sequence tags corresponding to T-DNA left borders (LB). For line *bt1*, information is from Kirchberger et al. (46).

^‡^ T-DNA lines are: emb2024: CS16134; emb3119-1: RATM11-0136-1H; emb3119-2: SAIL_874_E07; emb2728-2: SAIL_240_G08; emb1144: CS16193.

^§^ T-DNA LBs in *pgl3-3* were identified at +215 and +437 nucleotides relative the ATG start codon, likely due to tandem insertion of at least 2 T-DNAs.

^¶^ NA: not assigned; the T-DNA is inserted at −107 nucleotides relative the ATG start codon of *AtPGD1*. This line was not studied further.

^#^ In *rpe3-3* the T-DNA LB was identified at +147 nucleotides relative the ATG start codon. The insertion results in the substitution by T-DNA of ∼250 nucleotides of the *AtRPE3* genomic sequence between +147 and +398 nucleotides relative the ATG start codon (*SI Appendix*, Fig. S1).

### Abortion of *pgl3* Embryos Is Not Due to Toxicity.

Oxidation of Glc6P by Glc6P dehydrogenase (step 5 in Fig. 1) produces δ-6-phosphogluconolactone—the substrate for PGL (29)—that can convert nonenzymatically to the γ-form of the lactone. Both 6-phosphogluconolactones are electrophilic and can potentially react with intracellular nucleophiles, leading to structural and catalytic alterations of proteins and to cell toxicity (30). If loss of plastidial PGL3 results in accumulation of 6-phosphogluconolactones, then *pgl3* embryo lethality could be due to the toxicity of these compounds. To test this we identified mutant plants lacking plastidial 6-phosphogluconate dehydrogenase (PGD), which catalyzes the step of the OPPP beyond PGL (step 7 in Fig. 1). Such mutants would be defective in the OPPP but able to hydrolyze 6-phosphogluconolactones via PGL3, thus preventing accumulation of these toxic intermediates.

Two genes in *Arabidopsis*, *PGD1* (At1g64190) and *PGD3* (At5g41670), encode enzymes with dual plastidial and cytosolic location (31). A third gene, *AtPGD2* (At3g02360), encodes a cytosolic PGD that is also targeted to peroxisomes. Publicly available transcript data (32) show that *PGD1* and *PGD3* are highly expressed in all seed tissues during seed development (*SI Appendix*, Fig. S5*A*). *PGD2* transcript levels are much lower than those of *PGD1* and *PGD3* early during seed development; however, at later developmental stages all *PGD* genes are expressed at comparable levels in the seed. All 3 PGDs can form both homo- and heterodimers. Interaction of PGD1 or PGD3 with PGD2 does not target either PGD1 or PGD3 to peroxisomes or PGD2 to plastids; PGD2 is always excluded from plastids and enters peroxisomes following homodimer formation in the cytosol (33). Hence, PGD1 and PGD3 account for most or all of the plastidial PGD activity.

We identified homozygous *pgd1* and *pgd3* mutants in progeny from independent T-DNA insertion lines (Table 1 and *SI Appendix*, Fig. S1). *PGD1* and *PGD3* transcripts were absent from the *pgd1-1* and *pgd3-1* mutants, respectively; thus these mutants carry null alleles (*SI Appendix*, Fig. S6 *A* and *C*). *PGD1* transcript abundance was comparable to or slightly lower than that of wild-type plants in a second *pgd1* mutant, *pgd1-2*, in which the T-DNA is inserted 19 nucleotides upstream of the TGA stop codon (Table 1 and *SI Appendix*, Fig. S6*B*). Mutant *pgd1* and *pgd3* plants were normal and seed development was similar to that of wild-type plants (*SI Appendix*, Table S1). Hence, loss of only 1 plastidial PGD does not compromise normal growth and viability.

We crossed *pgd3-1* with *pgd1-1*. No *pgd1;pgd3* mutant plants were identified in progeny of selfed *+/pgd1;+/pgd3* parental lines and segregation of each mutant allele was distorted from the expected ratio for 2 cosegregating T-DNA insertions (*SI Appendix*, Table S2). Developing seeds in siliques on either *+/pgd1;−/pgd3* or *−/pgd1;+/pgd3* plants (Fig. 2*D* and *SI Appendix*, Fig. S7) fell into 4 phenotypic classes: (*i*) phenotypically normal green seeds, (*ii*) abnormal white seeds that collapsed later during development, (*iii*) small brown seeds that arrested early in development, and (*iv*) unfertilized or aborted ovules. Green seeds segregated at a ratio of about 1:1 with abnormal or arrested seeds and ovules (*SI Appendix*, Table S1), suggesting that complete loss of plastidial PGD activity results in embryo abortion. In addition, the presence of unfertilized or aborted ovules indicates that the mutations are to some extent gametophyte lethal.

Embryos from white seeds aborted early during development. When phenotypically normal seeds contained embryos at the late torpedo or green cotyledon stages, white seeds contained embryos that arrested at the preglobular to globular stage (Fig. 2 *E*–*G* and *SI Appendix*, Fig. S7). Small brown seeds that had already collapsed often contained preglobular-stage embryos (Fig. 2 *F* and *G* and *SI Appendix*, Fig. S7). Embryos aborting at the globular stage were raspberry-like: cells in the embryo proper had proliferated by unorganized cell divisions and suspensors often had multiple cell layers (Fig. 2*E* and *SI Appendix*, Fig. S7).

Similar defects were seen in seeds of plants generated by crossing *pgd3-1* with *pgd1-2* (*SI Appendix*, Fig. S7 *L* and *O*), except that these plants produced some embryos that progressed through the globular stage and aborted at the heart-early torpedo stage (*SI Appendix*, Fig. S7 *N* and *Q*), consistent with the presence of *PGD1* transcript in the *pgd1-2* mutant. The presence of residual PGD1 activity in the absence of PGD3 thus may allow progression beyond the globular stage but is not sufficient for development to maturity. Overall these results show that loss of both *PGD1* and *PGD3* is lethal at or before the globular stage. The similarity of this phenotype and that of *pgl3* is consistent with a requirement for the full oxidative part of the plastidial OPPP, arguing strongly against the possibility that abortion in *pgl3* mutants is due to accumulation and toxicity of 6-phosphogluconolactones in these mutants. Collectively, our studies of *gpt1* (9), *pgl3*, and *pgd* mutants show that Glc6P metabolism to Ru5P through the oxidative section of the OPPP is essential for embryo morphogenesis. Loss of this flux results in developmental arrest at the globular stage and the formation of raspberry-like embryos.

### Two Routes That Potentially Bypass the Oxidative Part of the OPPP Are Not Required for Development through the Globular Stage.

The oxidative part of the plastidial OPPP generates Ru5P, the substrate for the nonoxidative part of the pathway. Two other routes could also generate intermediates of the nonoxidative part, thus potentially bypassing the oxidative part of the OPPP. First, transketolase (TKL) (step 10 in Fig. 1) can use glyceraldehyde 3P and Fru6P to generate erythrose-4-phosphate (E4P), Xyl5P, and hence R5P (26, 34, 35). Both substrates are available inside the plastid: Fru6P can be generated via plastidial phosphoglucose isomerase (step 12 in Fig. 1) and glyceraldehyde 3P can be imported via the plastid envelope triose phosphate transporter (step 4 in Fig. 1) or generated from Fru6P in plastidial glycolysis (Fig. 1). Second, the plastid envelope Xyl5P transporter (XPT) (step 2 in Fig. 1) can import Ru5P and Xyl5P from the cytosol (13, 14).

Two *Arabidopsis* genes encode plastidial TKL. *TKL1* (At3g60750) is expressed throughout embryo development and likely encodes the major TKL isoform of the embryo (*SI Appendix*, Fig. S5*B*) (32). *TKL2* (At2g45290) is expressed mainly in the seed coat and transcript levels are much lower than those of *TKL1* in the embryo at the globular and heart stages. Homozygous *tkl1* mutants were absent in progeny from 2 independent T-DNA insertion lines, *tkl1-1* and *tkl1-2* (Table 1 and *SI Appendix*, Fig. S1). Genotyping of progeny of selfed +/*tkl1* plants confirmed that the mutations were recessive and lethal (*SI Appendix*, Table S2). Siliques of *+/tkl1* plants contained about one-quarter white seeds, which collapsed before seed maturity (Fig. 2*H* and *SI Appendix*, Fig. S2*C* and Table S1). Although these seeds were not viable, they contained embryos that progressed beyond the globular stage before seed abortion (Fig. 2*I* and *SI Appendix*, Fig. S2*E*). Delayed development of embryos in white seeds was apparent by 6 DAF (*SI Appendix*, Fig. S4*D* and Table S4), when 30% of embryos were still at the heart-early torpedo stage, whereas 90% of the embryos in wild-type seeds and phenotypically normal green seeds on *+/tkl1* plants were at torpedo stage. Embryo development in white seeds progressed beyond this stage at a slow rate, reaching torpedo stage and then arresting when 68% of embryos in green seeds were already at the upturned-U or expanded cotyledon stages of development (*SI Appendix*, Fig. S4*D* and Table S4).

To test whether XPT is required for normal embryo development, we identified homozygous *xpt* mutants in progeny from 2 independent T-DNA insertion lines, SAIL_378_C01 (x*pt-2*) (36) and GT_5_112515 (*xpt-4*) (Table 1 and *SI Appendix*, Fig. S1). No *XPT* transcript was detected in the *xpt-2* mutant, suggesting that it carries a null allele (*SI Appendix*, Fig. S6*D*). The phenology, reproduction, fecundity, and seed development of both *xpt* mutants were indistinguishable from those of wild-type plants (*SI Appendix*, Table S1). *XPT* transcript levels are lower than *GPT1* in most seed tissues during embryogenesis, but levels in the embryo are higher or at least comparable to those of *GPT1* from the preglobular until the torpedo stage of development (*SI Appendix*, Fig. S5*C*). The lack of an observable phenotype in *xpt* embryos thus is unlikely to be due to lack of *XPT* expression during early development.

We checked whether TKL and XPT exhibit mutual redundancy with respect to embryo development through the globular stage, by examining embryos on *+*/*tkl1-1;xpt-2* plants from crosses between *xpt-2* and *+*/*tkl1-1*. No *XPT* transcript was detected in these plants (*SI Appendix*, Fig. S6*D*). Siliques on +/*tkl1-1;xpt-2* plants contained 22% white seeds in which embryos progressed through the globular stage but arrested at torpedo stage before abortion, as for embryos in white seeds of *+/tkl1* plants (Fig. 2*J* and *SI Appendix*, Table S1). Thus, the TKL and XPT routes for generation of intermediates of the nonoxidative part of the OPPP are not required for development through the globular stage, either individually or together.

We also checked whether loss of XPT would further compromise embryo development in embryos lacking PGL3. Plants with the *+*/*pgl3-3;xpt-2* genotype were identified from crosses between *+*/*pgl3-3* and *xpt-2*. No *XPT* transcript was detected in these plants (*SI Appendix*, Fig. S6). Their siliques contained about 25% white seeds in which embryos progressed only to globular stage, then formed raspberry-like structures (Fig. 2*C* and *SI Appendix*, Table S1), as for embryos in white seeds of *+/pgl3-3* plants.

Overall, these results show that TKL and XPT are not required for provision of intermediates of the nonoxidative part of the OPPP during development through the globular stage. One or both pathways may carry flux in wild-type plants, but these fluxes are either not appropriate or insufficient to bypass the requirement for the oxidative part of the OPPP. Although TKL is required for later stages in embryo development, it is largely or wholly redundant for progression through the globular stage.

### Embryos Defective in Plastidial R5P Generation Abort before the Globular Stage.

Intermediates of the nonoxidative part of the OPPP are substrates for major biosynthetic pathways. R5P is the immediate substrate for purine synthesis (37), and E4P is the substrate for generation through the shikimate pathway of chorismate, the precursor of biosynthesis of aromatic amino acids, and a host of primary and secondary metabolites derived from them (38, 39). It is reasonable to suppose that products of both the purine and the shikimate pathways are required during embryo development. Hence, the essential role of the oxidative part of the OPPP for development through the globular stage might reflect its importance in generating Ru5P as the precursor of intermediates of the nonoxidative section of the OPPP required for purine and shikimate synthesis.

To evaluate the importance of Ru5P generation via the oxidative part of the OPPP for purine nucleotide synthesis, we examined mutants defective in the interconversion of Ru5P and R5P, catalyzed by plastidial R5P isomerase (RPI) (step 8 in Fig. 1). Of the 4 *Arabidopsis* genes encoding RPI (26), *RPI3* (At3g04790), and *RPI4* (At5g44520) encode proteins with putative N-terminal transit peptides for plastidial localization. *RPI3* is expressed in the embryo proper and the syncytial endosperm at the preglobular stage, and expression levels increase in all seed tissues throughout development (*SI Appendix*, Fig. S5*E*) (32). *RPI4* is not expressed in preglobular embryos and transcript abundance is at or below basal levels in most seed tissues during early embryogenesis (this is also true for the genes encoding putatively cytosolic isoforms, RPI1 and RPI2) (*SI Appendix*, Fig. S5*D*). An *rpi4* mutant had no observable phenotype (40). The RPI4 protein lacks 2 of the 3 residues required for catalysis in RPI-like proteins (40–42) and has low amino acid sequence similarity compared with the other RPIs. It may therefore have a different biochemical function from other RPIs (40). It is thus highly likely that plastidial RPI activity in early embryos is attributable to RPI3.

**Fig. 4.**
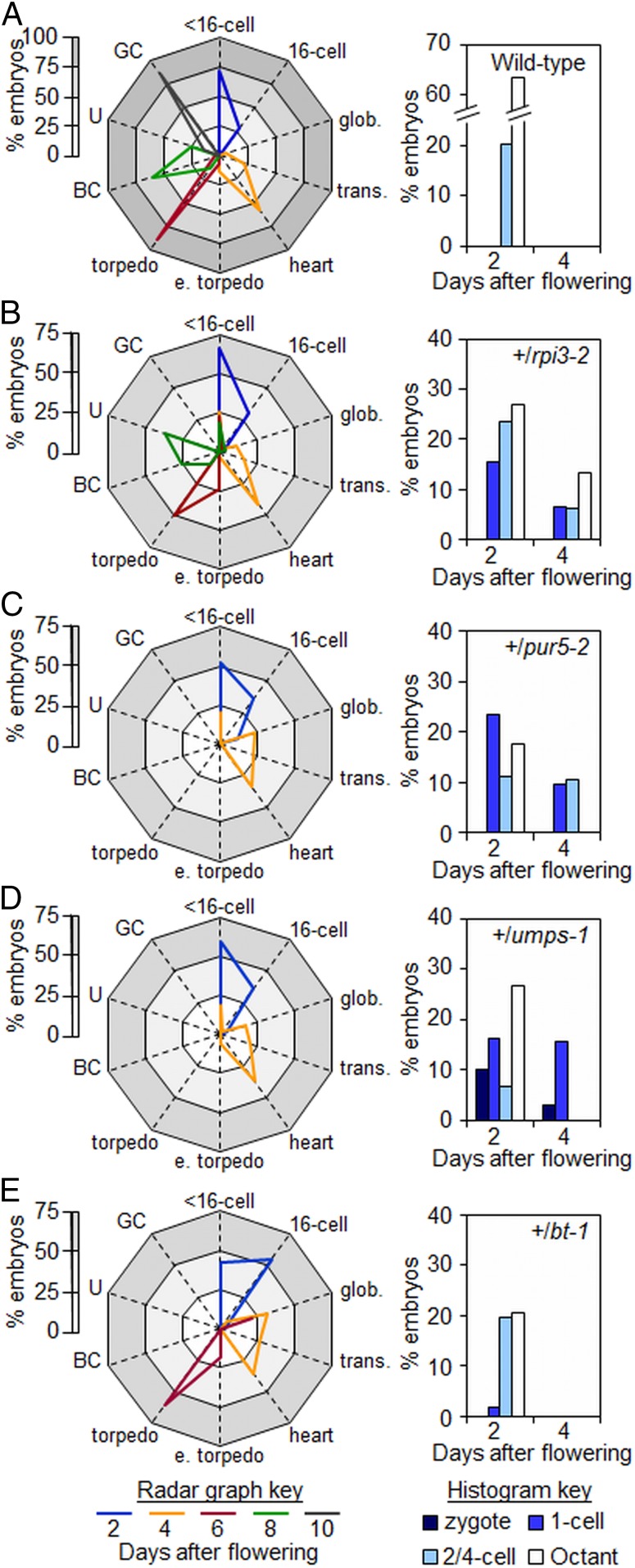
Developmental progression of embryos from wild-type and heterozygous plants. (*Left*) Radar graphs showing the developmental stages of embryos at particular time points (key at the base of the figure). Within each graph the white zone represents 0–25%, the light gray zone 25 to 50%, and the gray zone 50–75% of embryos examined. In *A* the dark gray zone represents 75 to 100% of embryos examined (for clarity the scale is 0 to 100% in *A* and 0 to 75% in *B*–*E*). (*Right*) Histograms of the distribution of embryos at preglobular stages of development (referred to as <16 cells in the radar graphs) at 2 and 4 DAF. Seeds were from (*A*) wild-type, (*B*) +/*rpi3-2*, (*C*) +/*pur5-2*, (*D*) +/*umps-1*, (*E*) +/*bt-1* plants. Abbreviations: BC, bent-cotyledon; e. torpedo, early torpedo; GC, green cotyledon; glob., globular; trans., transition; U, upturned-U. See *SI Appendix*, Table S4 for original data.

The SeedGenes collection of embryo-lethal mutants (http://seedgenes.org/) (4) contains 2 insertion lines with mutations in *RPI3* that are reported to cause early embryo arrest, emb3119-1 and emb3119-2 (hereafter referred to as *rpi3-1* and *rpi3-2*, respectively) (Table 1 and *SI Appendix*, Fig. S1). Homozygous *rpi3* mutants were absent from progeny from these lines (Table 1 and *SI Appendix*, Fig. S1 and Table S2). Siliques on *+*/*rpi3* plants contained about 25% white seeds that turned brown and collapsed early during seed development (Fig. 2*K* and *SI Appendix*, Table S1). Embryo defects in seeds destined for abortion were apparent by 4 DAF. Whereas 95% of wild-type embryos were at transition to early-torpedo stages, more than 20% of embryos from *+/rpi3-2* plants were still at the preglobular stage and did not progress beyond the octant stage (see Figs. 2*L* and 4*B*, and *SI Appendix*, Figs. S2 *F* and *G* and S8 *E*–*H*). In wild-type embryos following the first zygotic division, the apical daughter cell undergoes 2 rounds of longitudinal divisions to form a 2-cell then a 4-cell embryo (43). In contrast, almost 20% of the seeds destined for abortion from +/*rpi3* plants contained embryos in which the first division of the apical daughter cell was longitudinal but the second was transverse (*n* = 58 seeds examined) (arrows in Fig. 2*M* and *SI Appendix*, Fig. S8 *F*–*H*). In addition, 3% of the *rpi3* embryos underwent a transverse rather than longitudinal division at the 1-cell stage, forming a 2-cell embryo proper with incorrectly positioned division planes (arrow in Fig. 2*N*). These results show that conversion of Ru5P to R5P via RPI3 in the plastid is essential for correct pattern formation and seed viability.

Full operation of the nonoxidative part of the OPPP requires both R5P and Xyl5P as substrates for TKL. Our data thus far show that R5P production is essential for progression beyond the globular stage of embryo development, but Xyl5P production may not be essential. Embryos lacking 2 possible routes for Xyl5P production—TKL and the XPT—progress beyond the globular stage. However, Xyl5P may be produced by a third route, from Ru5P via the only plastidial isoform of Ru5P epimerase (RPE3) (step 9 in Fig. 1). Loss of RPE3 is reported either to result in embryo arrest at the cotyledon and later stages (http://seedgenes.org/, emb2728) or to be seedling-lethal (44). We examined 2 mutants with insertions in *RPE3*: *rpe3-2* (emb2728-2) and *rpe3-3* (SAIL_240_G08) (Table 1 and *SI Appendix*, Fig. S1). Homozygous *rpe3* mutants were absent from progeny of these 2 lines, implying that loss of *RPE3* expression is lethal. Siliques on *+/rpe3-3* plants contained about one-quarter pale-green seeds (*SI Appendix*, Table S1) in which embryos reached at least the torpedo stage and could grow to fill the seed, but did not acquire the U-shape of normal embryos (*SI Appendix*, Fig. S8 *O* and *P*). Therefore, RPE3 is not required for development through the globular stage. Overall, these results suggest that the essential function of the OPPP in early embryo development is the production specifically of R5P, and that generation of Xyl5P may not be required.

### Purine Synthesis Is Essential for Embryo Morphogenesis.

To investigate whether the requirement for generation of R5P via the oxidative part of the plastidial OPPP stems from consumption of R5P (for nucleotide synthesis) or of E4P (for shikimate synthesis), we examined the effect on embryo development of mutations directly affecting enzymes on the pathways of either shikimate or nucleotide synthesis. It seems unlikely that the shikimate pathway is essential for early embryo development. A T-DNA insertion predicted to disrupt expression of chorismate synthase (*CS*; At1g48850, catalyzing the conversion of 5-enolpyruvylshikimate-3-phosphate to chorismate) (step 17 in Fig. 1), was embryo-lethal but did not prevent embryo development through the globular stage (emb1144, http://seedgenes.org/; hereafter referred to *cs-1*). Homozygous *cs-1* mutants were absent from progeny of heterozygous plants. More than 20% of seed from siliques of +/*cs-1* plants contained embryos arrested at the torpedo stage, when sibling embryos were approaching maturity (see Fig. 3*K* and *SI Appendix*, Table S1), suggesting that chorismate synthesis is not essential for embryo morphogenesis. Thus, the requirement for the oxidative part of the OPPP beyond the globular stage does not stem from a role in providing E4P for chorismate synthesis.

In the first step of nucleotide synthesis, R5P in the plastid is converted to the activated ribose precursor 5-phosphoribosyl-1-pyrophosphate (PRPP) (37). Eleven plastidial enzymes convert PRPP to purine nucleotides, which are exported from the plastid (Fig. 3*A*). If R5P generation is essential because it is a substrate for purine synthesis, then mutations affecting purine synthesis downstream of R5P should result in early embryo arrest. To test this, we examined the effect on embryo development of the loss of 2 plastidial proteins on the pathway of purine synthesis and subsequent nucleic acid synthesis: phosphoribosyl-formylglycinamidine cyclo-ligase (PUR5) and the plastidial adenine nucleotide transporter (BRITTLE1; BT1).

PUR5 converts phosphoribosyl-formylglycinamidine and glycine into 5-aminoimidazole ribonucleotide (AIR) (step 15 in Fig. 3*A*) (37, 45). We obtained independent lines with T-DNA insertions in the *PUR5* gene (At3g55010), which are reported to be embryo-lethal (http://seedgenes.org/; emb2818-1 [SALK_070673] and emb2818-2 [SAIL_343_A07], hereafter referred to as *pur5-1* and *pur5-2*, respectively) (Table 1 and *SI Appendix*, Fig. S1). Homozygous *pur5* mutants were absent from progeny of *+/pur5* plants (*SI Appendix*, Table S2). Siliques on *+/pur5* plants contained some very small transparent seeds that turned brown and collapsed by 4 DAF (Fig. 3 *B*–*D* and *SI Appendix*, Table S1). On +/*pur5-1* plants, the ratio of abnormal to normal seeds was statistically significantly less than the expected 1:3, indicating that some mutants aborted as zygotes or that the mutation was to some extent gametophyte-lethal (*SI Appendix*, Table S1). Embryos in seeds destined for abortion were defective by 2 DAF: almost 25% had not progressed beyond the 1-cell stage (Fig. 4*C* and *SI Appendix*, Fig. S8). By 4 DAF *pur5* embryos remained at the 1-cell or 2-cell stages (47% and 53%, respectively, *n* = 44) (Fig. 3 *C* and *D* and *SI Appendix*, Figs. S2*H* and S8 *I*–*L*). The embryo phenotype of *pur5* mutants is thus more severe than that of *rpi3* mutants unable to generate R5P in the plastid.

The BT1 transporter (step 16 in Figs. 1 and 3*A*) facilitates export from the plastid of newly synthesized purines in the form of adenine nucleotides. Its loss causes seed abortion, with embryo arrest before morphogenesis (46, 47). Using a previously described T-DNA insertion mutant (At4g32400; emb104-3) (46, 47) we found that siliques on *+/bt1* plants contained about 25% white seeds that collapsed later in development (Fig. 3*E*). Whereas more than 70% of wild-type embryos were at heart-early torpedo stage by 4 DAF, almost 40% of embryos from *+/bt1* plants were at the globular stage (Fig. 4 *A* and *E* and *SI Appendix*, Fig. S3). Like *gpt1* (9) and *pgl3* mutants, embryos in seeds destined for abortion arrested at the globular stage and were raspberry-like. Early embryo development thus requires both purine synthesis from R5P in the plastid and the export of the adenine nucleotide products of this pathway to the cytosol.

Purine synthesis may be essential for embryo morphogenesis because it is required for nucleic acid synthesis, but its importance might alternatively or additionally reflect a requirement for cytokinin synthesis at early developmental stages. Cytokinins are synthesized predominantly inside plastids, from purines (AMP, ADP, ATP) and intermediates of the plastidial methylerythritol phosphate pathway of isoprenoid synthesis (48). The fact that export of adenine nucleotides via BT1 is essential for morphogenesis suggests that the requirement for purines is for nucleic acid rather than cytokinin synthesis. To provide further insight into whether nucleic acid synthesis is essential for morphogenesis, we examined the effect on embryos of loss of an enzyme of pyrimidine synthesis, orotate phosphoribosyltransferase/orotidine-5P decarboxylase/UMP synthase (UMPSase) (step 18 in Fig. 3*G*). The pyrimidine pathway is independent of the purine pathway (its requirement for R5P is met by the cytosolic OPPP), it is not required for cytokinin synthesis, and apart from the first 2 steps, the pathway including UMPSase is cytosolic (37, 49, 50).

We identified a T-DNA insertion line in which expression of the gene encoding UMPSase, At3g54470, is predicted to be affected (Table 1 and *SI Appendix*, Fig. S1). Homozygous *umps-1* mutants were absent from progeny of heterozygous plants (*SI Appendix*, Table S2). The T-DNA segregated at a ratio statistically significantly lower than the expected 2:1 (T-DNA:wild-type) for an embryo-lethal allele. Developing siliques on +/*umps-1* plants contained some small, abnormal seeds that collapsed by 4 DAF (Fig. 3*H*). The ratio of abnormal to normal seeds was statistically significantly lower than the expected 1:3 (*SI Appendix*, Table S1). These results suggest low transmission of the mutant allele. Embryos in abnormal seeds arrested at the 1-cell stage (Figs. 3*I* and 4 *A* and *D*, and *SI Appendix*, Fig. S8); in some cases, zygotes had elongated but had not undergone the first asymmetric division along the apical-basal axis (Fig. 3*J* and *SI Appendix*, Fig. S8 *M* and *N*). Therefore, pyrimidine as well as purine synthesis is essential for early embryo development; hence, loss of nucleic acid synthesis is a primary cause of developmental arrest before morphogenesis.

### Defects in R5P Synthesis and the Purine and Pyrimidine Pathways Disrupt Endosperm Development.

*Arabidopsis* seeds attain their final size largely through rapid proliferation of the endosperm and cell divisions and expansion in the seed integuments (51, 52). Subsequent, relatively small increases in seed volume are mostly due to embryo expansion. White seeds in siliques on +/*pgl3-3* (Fig. 2*A*), +/*tkl1* (Fig. 2*H*), +/*bt1* and +/*cs-1* (Fig. 3 *E* and *K*) plants expanded before abortion even though the embryos they contained occupied only a small fraction of the internal volume. This implies that early endosperm development was not affected by loss of function of these genes, or was affected much less than embryo development. To test this, we examined endosperm development in seeds on +/*pgl3-3* plants. Endosperm development was the same in green seeds with embryos at the heart or torpedo stage and white-aborting seeds with globular or raspberry-like embryos (*SI Appendix*, Fig. S9 *A*–*F*). However, for 3 of the mutants, white seeds in siliques on heterozygous plants failed to expand before abortion (*+/rpi3-1*, *+/pur5-2*, +/*umps*-*1*) (Figs. 2*K* and 3 *B* and *H*).

**Fig. 5.**
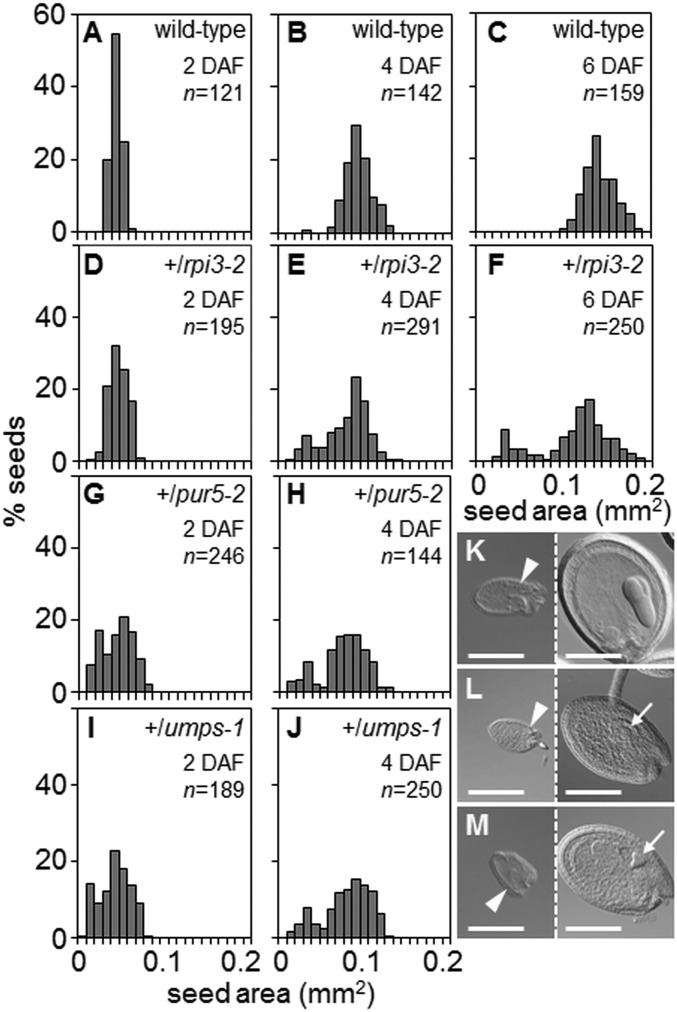
Seed size distribution. Seed areas were measured at 2, 4, and 6 DAF. Numbers (*n*) of seeds measured for each age and genotype are shown. (*A*–*C*) Wild-type, (*D*–*F*) +/*rpi3-2*, (*G* and *H*) +/*pur5-2*, and (*I* and *J*) +/*umps-1.* (*K*, *L*, and *M*) DIC images of aborting (*Left*) and normal (*Right*) seeds at 6 DAF from a silique on (*K*) +/*rpi3-2*, (*L*) +/*pur5-2*, (*M*) +/*umps-1* plants. The normal seeds in *L* and *M* contain a heart-stage embryo (arrows). Arrowheads: micropylar end of the seeds. (Scale bars, 200 μm.)

**Fig. 6.**
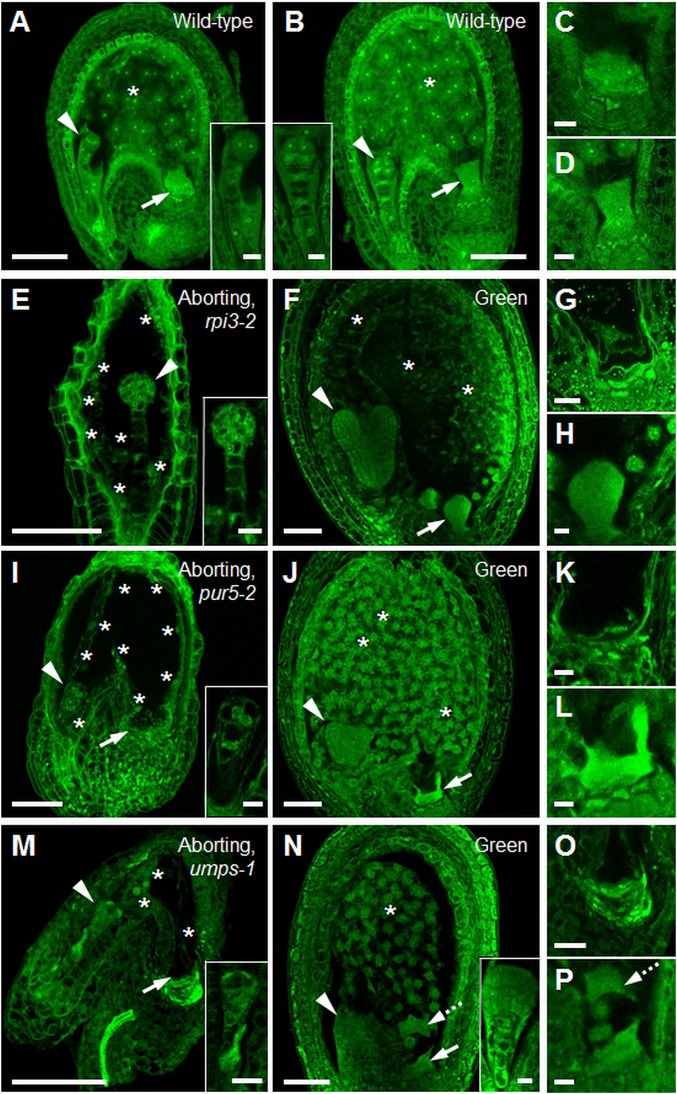
Endosperm development in seeds destined for abortion. (*A*) Wild-type seed with embryo at the 2-/4-cell stage (*Inset*) and (*B*) at the 8-cell stage (*Inset*). (*C*) Close-up of the chalazal cyst from *A* and (*D*) from *B*. The chalazal cyst is normal. (*E*) Aborting seed from a +/*rpi3-2* plant with embryo arrested at the 8-cell/dermatogen stage (*Inset*). (*F*) Green seed from the same silique as in *E*, with torpedo-stage embryo. The endosperm is cellularized. (*G*) Close-up of the chalazal-end of an aborting seed from a *+/rpi3-2* plant. The chalazal cyst failed to develop. (*H*) Close-up of the chalazal cyst in *F*. The chalazal cyst is normal. (*I*) Aborting seed from a +/*pur5-2* plant with embryo arrested at the 2-/4-cell stage (*Inset*). (*J*) Green seed from the same silique as in *I*, with a heart-stage embryo. (*K*) Close-up of the chalazal end of the seed in *I*. The chalazal cyst failed to develop. (*L*) Close-up of the chalazal cyst in *J*. The chalazal cyst is normal. (*M*) Aborting seed from a +/*umps-1* plant with embryo arrested at the elongated zygote/1-cell stage (*Inset*). (*N*) Phenotypically normal seed from the same silique as in *M* with a late-globular stage embryo. Endosperm development is normal. (*O*) Close-up of the chalazal end of the seed in *M*. The chalazal cyst failed to develop. (*P*) Close-up of the chalazal cyst of the seed in *N*. The chalazal cyst is normal. Dashed arrows in *N* and *P* show part of the chalazal cyst that was detached from its original position during sample preparation. Throughout, arrowheads point to embryos, arrows to the chalazal cyst, asterisks indicate endosperm nuclei. Seeds were Feulgen-stained and imaged under a confocal microscope. Images of whole seeds are single (*F*) or maximum optical projections of (*A*) 15, (*B*) 8, (*E*) 19, (*I*) 38, (*J*) 10, (*M*) 14, (*N*) 30 *z*-slices covering the seed cavity. Images in *C*, *D*, *G*, *H*, *K*, *L*, *O*, and *P* are from a single *z*-slice. (Scale bars: *A*, *B*, *E*, *F*, *I*, *J*, *M*, and *N* are 50 μm; and *C*, *D*, *G*, *H*, *K*, *L*, *O*, and *P* and *Insets* in *A*, *B*, *E*, *I*, *M*, and *N* are 10 μm.)

For wild-type plants, seed cross-sectional area increased progressively from 2 to 6 DAF, with an approximately normal distribution of areas (Fig. 5 *A*–*C*). In contrast, the distribution of seed areas for *+/rpi3-1*, *+/pur5-2*, and +/*umps-1* became increasingly bimodal from 2 to 6 DAF (Fig. 5). At 6 DAF, seeds in the smaller peak (about 20% of the total) contained preglobular-stage embryos, whereas embryos in seeds in the larger peak had progressed to the torpedo stage (Fig. 5). We investigated whether the failure of expansion in seeds destined for abortion in these mutants correlated with defects in endosperm development. In wild-type seeds with embryos at the 2-/4-cell or octant stages, endosperm proliferation had formed a syncytium with evenly distributed nuclei (Fig. 6 *A* and *B* and Movies S1 and S2). All 3 mutations caused severe endosperm proliferation defects. In seeds destined for abortion from *+/rpi3-2* and *+/pur5-2* plants, the endosperm underwent initial mitotic divisions to form a syncytium-like domain, but the peripheral endosperm had many fewer nuclei than in wild-type seeds of comparable developmental stage and nuclei were unevenly distributed along the anterior-posterior axis (Fig. 6 *E* and *I*, *SI Appendix*, Fig. S9 *G* and *H*, and Movies S3 and S4). In phenotypically normal siblings, the endosperm had completed the syncytial phase of development, and was cellularizing at the anterior and peripheral domains of the syncytium (Fig. 6 *F* and *J*). In aborting seeds on +/*umps-1* plants, endosperm proliferation ceased at or immediately after the first mitotic division, and nuclei were enlarged (Fig. 6*M*, *SI Appendix*, Fig. S9*I*, and Movie S5). In all 3 mutants the chalazal cyst did not form at the posterior pole (*rpi3-2* in Fig. 6*G*, *pur5-2* in Fig. 6 *I* and *K*, and *umps-1* in Fig. 6 *M* and *O*). No defects in chalazal cyst development were apparent in phenotypically normal siblings (Fig. 6 *F*, *J*, and *N*). Thus, endosperm as well as embryo development is blocked specifically by defects directly affecting nucleotide synthesis, in the final step of plastidial R5P generation and in the purine and pyrimidine synthesis pathways.

## Discussion

Our results and those of Andriotis et al. (9) together show that progression of embryo development through the globular stage requires production of R5P inside the plastid via the oxidative section of the plastidial OPPP and RPI3. Loss of either Glc6P import or plastidial PGL3 or PGD results in developmental arrest at the globular stage and a distinctive, raspberry-like terminal phenotype. Loss of RPI3, and hence conversion of the Ru5P product of the oxidative part of the pathway to R5P, results in even earlier embryo arrest at the 2-/4-cell stage. Conversely, progression of development through the globular stage does not require further metabolism of R5P in the OPPP. Development beyond the globular stage occurs in the absence of proteins that would permit R5P metabolism to other OPPP intermediates (TKL and XPT) and in the absence of Xyl5P generation from R5P (via RPE3).

Although R5P could in theory be generated via other routes, none of these can compensate for defects in the conversion of Glc6P to Ru5P via the oxidative part of the OPPP. Neither the import of pentose phosphates from the cytosol via XPT nor plastidial interconversion of phosphorylated sugars by TKL1 is essential for development through the globular stage; flux through XPT and TKL1 is not appropriate or not sufficient to support continued development in the absence of the oxidative part of the OPPP. It seems likely that in wild-type embryos, most or all of the substrate for R5P generation is provided through the oxidative part of the OPPP.

We propose that embryo lethality due to loss of R5P synthesis via the OPPP is attributable to failure to synthesize purines, and probably also histidine. In addition to its metabolism via the OPPP, the major fate for R5P inside plastids is conversion to PRPP, a substrate required for the synthesis in the plastid of both purines and histidine. Previous work showed that these PRPP-utilizing pathways are essential for embryo development from the earliest stages (2, 53), and our work extends these observations for purine synthesis. Embryos deficient in PUR5, the enzyme that catalyzes the fourth step of the pathway from R5P, arrested at the 1- or 2-cell stage. Loss of BT1, which transports newly synthesized purines from the plastid into the cytosol (46), resulted in arrest at the globular stage and the formation of raspberry-like embryos. In addition to purine nucleotide and histidine synthesis, conversion of R5P to AIR through PUR5 is also required for the synthesis of thiamine (vitamin B1). A chloroplast-localized iron-sulfur cluster protein THIC catalyzes the first step in the conversion of AIR to 2-methyl-4-amino-5-hydroxymethyl pyrimidine diphosphate, which is coupled to thiazole to form thiamine monophosphate (54). The best-characterized *thiC* mutant retains about 10% of wild-type levels of *THIC* transcript. Viable seeds are produced but seedlings are albino and fail to establish (54, 55). Thiamine synthesis is thus essential for seedling growth, but it is not clear whether and at what stage of embryo development a loss of AIR synthesis from R5P might become limiting for thiamine synthesis and thus lead to developmental arrest.

The purine ATP is essential for cellular metabolism in its own right, and as the starting point for synthesis of nucleic acids and the hormone cytokinin. We suggest that a block in nucleic acid rather than in plastidial cytokinin synthesis is the primary reason for embryo developmental arrest when purine synthesis or the OPPP is blocked. First, export of adenine nucleotide to the cytosol via BT1 is essential at the globular stage of development. Second, synthesis of the pyrimidine, as well as the purine component of nucleic acids, is essential at the preglobular stage.

Our results suggest that loss of capacity for nucleic acid synthesis directly affects early endosperm development as well as the embryo. In seeds containing *rpi3*, *pur5*, or *umps* mutant embryos, cessation of nuclear proliferation in the endosperm occurred at a developmentally earlier time point than cessation of embryo development. When wild-type embryos are at the 2-/4-cell stage, the surrounding endosperm contains 44 to 48 nuclei (56, 57). When developmental arrest occurred in *rpi3*, *pur5*, or *umps* embryos, between the 2- and 8-cell stage, the surrounding micropylar and peripheral endosperm had undergone many fewer divisions than in a wild-type seed (Fig. 6). The chalazal cyst (a structure important for transfer of nutrients from maternal to filial tissues) (57) did not develop, and seeds were smaller than wild-type seeds at the same stage of embryo development. Such retarded development of the endosperm would not be expected if cessation of endosperm development were simply a downstream consequence of the arrest of embryo development.

If the plastidial OPPP is the sole route of substrate supply for purine synthesis, the phenotypes of embryos lacking components of the oxidative part of the OPPP or RPI3 would be expected to be the same as those of embryos lacking PUR5. This was not the case; developmental arrest occurred at the 2-/4-cell stage in *rpi3* and *pur5* mutants, but not until the globular stage in mutants deficient in GPT1 (9) or PGL3. This indicates that although most of the Ru5P for R5P, and hence purine synthesis, comes from the oxidative part of the OPPP, there are additional, minor routes of Ru5P generation in the plastid. These might include import via XPT, or generation via RPE from Xyl5P. Partial redundancy of routes may also explain why *bt1* embryos arrest later than *pur5* embryos. Although BT1 is by far the major means for export of purines from the plastid (46), it is possible that small amounts can be exported via other plastid envelope transporters.

The requirement for the oxidative section of the OPPP beyond the globular stage does not stem from a role in provision of precursors for aromatic amino acid synthesis. Mutants defective in chorismate synthesis did not undergo developmental arrest until the torpedo stage of embryo growth, and neither RPE nor TKL—enzymes of the nonoxidative part of the OPPP involved in synthesis of the chorismate pathway precursor E4P—were required for progression of embryos through the globular stage. The fact that chorismate synthase (*cs-1*) and *tkl1* mutants both undergo arrest at torpedo stage may indicate that progress beyond the torpedo stage requires generation of E4P specifically via the nonoxidative part of the OPPP. Before this point, aromatic amino acids may be supplied from surrounding tissues or alternative pathways may generate E4P in the plastid. For example, transaldolase could catalyze synthesis of E4P from Fru6P and glyceraldehyde 3P (step 11 in Fig. 1), or E4P could be imported from the cytosol into the plastid. Some plastid envelope transporters can transport E4P, including GPT1 and XPT (13). However, there is no established pathway of synthesis of E4P in the cytosol since neither TKL nor transaldolase are believed to be present in this compartment (26).

Our results show that the OPPP is required for normal pattern formation in the *Arabidopsis* embryo, in addition to its essential role in embryo growth. In wild-type embryos a precise series of cell divisions at the preglobular and globular stages establishes the apical-basal axis, radial symmetry, and the basic body plan (43, 58). Two longitudinal divisions of the apical daughter cell formed during the first zygotic division, followed first by transverse and then tangential divisions, specify the protoderm—the precursor of the epidermis—and separate it from the inner cells of the embryo proper (dermatogen stage embryo) (43). Root tissues and stem cells are specified during the globular stage through asymmetric division of the uppermost suspensor cell and a highly ordered pattern of basal embryo cell divisions. This pattern of cell divisions is not seen in embryos with blocks in either generation of R5P from imported Glc6P via the oxidative part of the plastidial OPPP, or purine synthesis and export. Although these embryos appear normal at the 1-cell stage, subsequent aberrant cell divisions result in abnormal morphologies. Depending on the position of the block, embryos either show early division abnormalities before or at the quadrant stage—thus failing to specify the protoderm and the precursors of the ground and vascular tissues—or undergo atypical divisions at a slightly later stage, resulting in an abnormal protoderm and failure to establish radial symmetry and basal body structures (“raspberry” embryos). Similar abnormalities are seen in *toz* mutants, which are defective specifically in longitudinal cell divisions in the early embryo (59). Phenotypes of *toz* mutants include aberrant cell-division planes, failure to establish normal radial patterning and to initiate bilateral symmetry, and failure to develop beyond the globular stage. Since the molecular basis of the *toz* phenotype—loss of a nucleolar protein TORMOZ—is very different from that of the OPPP mutants, this comparison suggests that defects in longitudinal division will prevent patterning and postglobular development whatever their cause.

In summary, our results provide strong evidence that embryo progression through the globular stage and endosperm development are completely dependent on an adequate supply of plastidial R5P, as the substrate for purine and hence for nucleic acid synthesis and probably also for histidine synthesis. An adequate supply of R5P depends on import of Glc6P and its metabolism via the oxidative part of the OPPP and RPI. Previous research has revealed that an unexpectedly large proportion of embryo-lethal mutations affect aspects of “housekeeping” plastidial metabolism (7, 60). Our research highlights the essential function of the plastidial OPPP in embryo morphogenesis: this primary metabolic pathway has a central developmental as well as a housekeeping role.

## Materials and Methods

### Plant Material.

*Arabidopsis* plants carrying mutant alleles are described in Table 1, and were grown in a controlled environment room as described in *SI Appendix*, *SI Materials and Methods*.

### Mutant Isolation and Gene-Expression Analysis.

Mutant genotyping by PCR with gene and T-DNA specific primers and semiquantitative RT-PCR are described in *SI Appendix*, *SI Materials and Methods*.

### Microscopy.

Analysis of embryo developmental progression and abortion phenotypes by differential interference contrast (DIC) optics, Feulgen staining, and fluorescence analysis of endosperm growth by confocal microscopy are detailed in *SI Appendix*, *SI Materials and Methods*.

## Supplementary Material

Supplementary File

Supplementary File

Supplementary File

Supplementary File

Supplementary File

Supplementary File
