# Supplementary material for: The plastidial pentose phosphate pathway is essential for postglobular embryo development in Arabidopsis
Source: Proc Natl Acad Sci U S A. 2019 Jul 11;116(30):15297–306. doi: 10.1073/pnas.1908556116 (PMC6660741; doi:10.1073/pnas.1908556116)
Supplement: Supplementary File [file pnas.1908556116.sapp.pdf]

Supplementary Information for

The plastidial pentose phosphate pathway is essential for post-globular embryo development in Arabidopsis

Vasilios M. E. Andriotis[a,b,1] and Alison M. Smith[a]

[a]John Innes Centre, Norwich Research Park, Norwich NR4 7UH, UK

[b]School of Natural and Environmental Sciences, Devonshire Building, Newcastle University, Newcastle-upon-Tyne, NE1 7RU, UK

[1]To whom correspondence should be addressed: vasilios.andriotis@newcastle.ac.uk

This PDF file includes:

SI Materials and Methods

Figs. S1 to S9

Tables S1 to S5

Captions for Movies S1 to S5

SI References

Other supplementary materials for this manuscript include the following Supporting Videos:

Movies S1 to S5

www.pnas.org/cgi/doi/10.1073/pnas.1908556116

SI Materials and Methods

Plant growth conditions

Seed of *Arabidopsis thaliana* plants carrying mutant alleles (Table 1) was sown on ¼-strength Murashige and Skoog agar plates, incubated at 4°C for 3 d in the dark then set to germinate with 16 h light at 20°C. Three-week-old plants were genotyped by PCR and plants carrying mutant alleles were transferred to soil and grown in a controlled environment room at 20°C with 12 h light and 250 µmol photosynthetically active radiation m^{-2} s^{-1}.

DNA and RNA extraction, PCR and semi-quantitative RT-PCR

All primers are described in Table S5. Genomic DNA was extracted from leaves with the DNeasy® Plant Mini Kit (Qiagen, www.qiagen.com/). PCR reactions were as previously described (1) except that annealing was at 56°C unless otherwise stated. Total RNA was extracted from either 2-week-old seedlings (for *pgd* mutants and the wild-type) or leaves of 5-week-old plants (for *xpt*, *pgl3;xpt* and *tkl1;xpt* mutants and the wild-type) using the RNeasy plant Mini Kit (Qiagen). RNA was treated with DNAse I (Sigma-Aldrich, www.sigmaaldrich.com/) and first-strand cDNA was synthesized with SuperScript II reverse transcriptase according to the manufacturer's instructions (Invitrogen, www.invitrogen.com/). Semi-quantitative RT-PCR reactions were as described (1) except that annealing was at 59°C. *TUB2* (tubulin beta chain 2; At5g62690) was used as the internal control.

Flanking sequence tags corresponding to T-DNA left borders were amplified with gene and T-DNA specific primers, cloned into pCR8®/GW/TOPO® vector (Invitrogen), and both strands sequenced with BigDye3 according to the manufacturer's instructions (ThermoFisher, www.thermofisher.com), with M13 primers as described previously (1).

Histological and fluorescence analyses

Flowers on the main flowering stem of at least five plants were tagged 3-4 d after the onset of anthesis, when plants had formed at least ~4 siliques. Developing seeds were dissected under a microscope and cleared with modified Hoyer's solution as described (2). In order to establish embryo developmental progression of seeds from plants carrying mutant alleles, seeds were taken from 4-8 siliques pooled from three plants. Whole-mount preparations were viewed with differential interference contrast (DIC) optics with a Leica DM6000 microscope. Images were analyzed with the Leica Application Suite 4.2 (www.leica-microsystems.com). Seed area was measured with ImageJ2 (http://imagej.net/ through the Fiji distribution (http://fiji.sc/)).

For Feulgen staining, siliques were fixed overnight in ethanol/acetic acid (3/1, aq. v/v) and processed as described (3) with some modifications. Briefly, following fixation, siliques were

washed with distilled water then treated with 5N HCl for 1 h. They were then stained with periodic acid-Schiffs reagent (Sigma-Aldrich) for 3 h followed by extensive washes with distilled water at 4°C. Stained siliques were incubated in 70%, 95% and 98% (aq. v/v) solutions, each for 10-min. Excess staining solution was removed by repeated washes in 100% (aq. v/v) ethanol. Siliques were rehydrated by 15-min washes in a series of ethanol solutions [95%, 90%, 85%, 75%, 50%, 35% (aq. v/v)] and seeds dissected under a microscope prior to resin treatment. Samples were incubated for 1 h in 1/1 (v/v) ethanol/LR White resin (London Resin). They were then embedded in LR White resin for 16 h at 60°C on microscope slides. Fuelgen-stained seeds were viewed with a Leica SP5 confocal microscope with emission at 488 nm and excitation at 490–622 nm. Image analysis of single-frame fluorescent images was with the Leica Application Suite 4.2. For generation of *z*-stacks optical slices were acquired at 0.5-nm intervals from the top of the seed coat to cover the whole seed cavity. The ImageJ2 software was used for analysis of *z*-stacks and maximum projection images from the number of *z*-slices stated in Fig. 6.

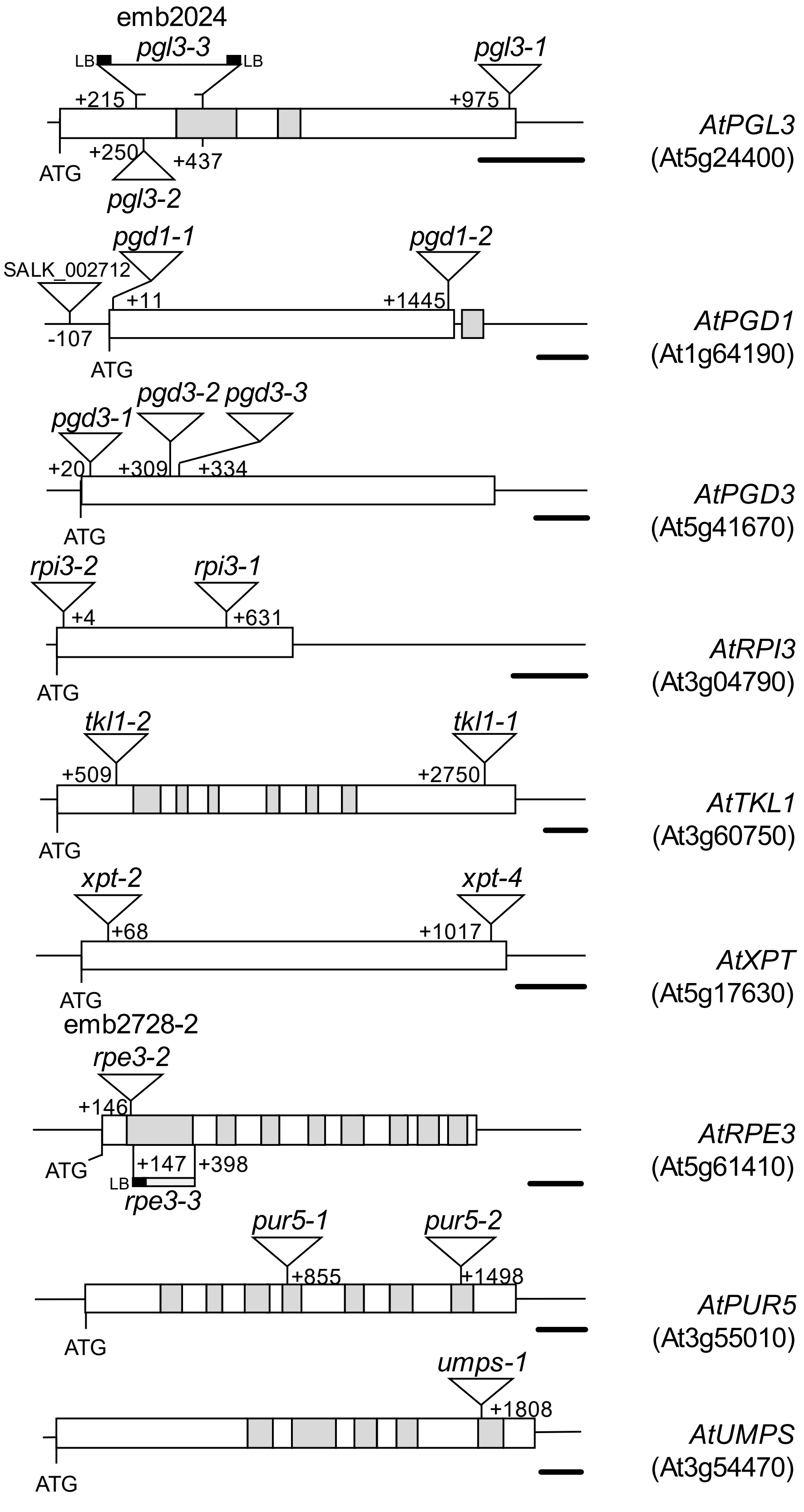

Fig. S1: Positions of insertions in T-DNA insertion lines used in this study. All insertions shown were confirmed by sequencing left border T-DNA flanking sequence tags. Open boxes represent exons and grey boxes are introns. Lines represent the 5'' and 3'UTR. The thicker lines below each gene model (on the right) are scale bars = 200 bp. Positions are indicated relative to the ATG start codon. See Table 1 for further details.

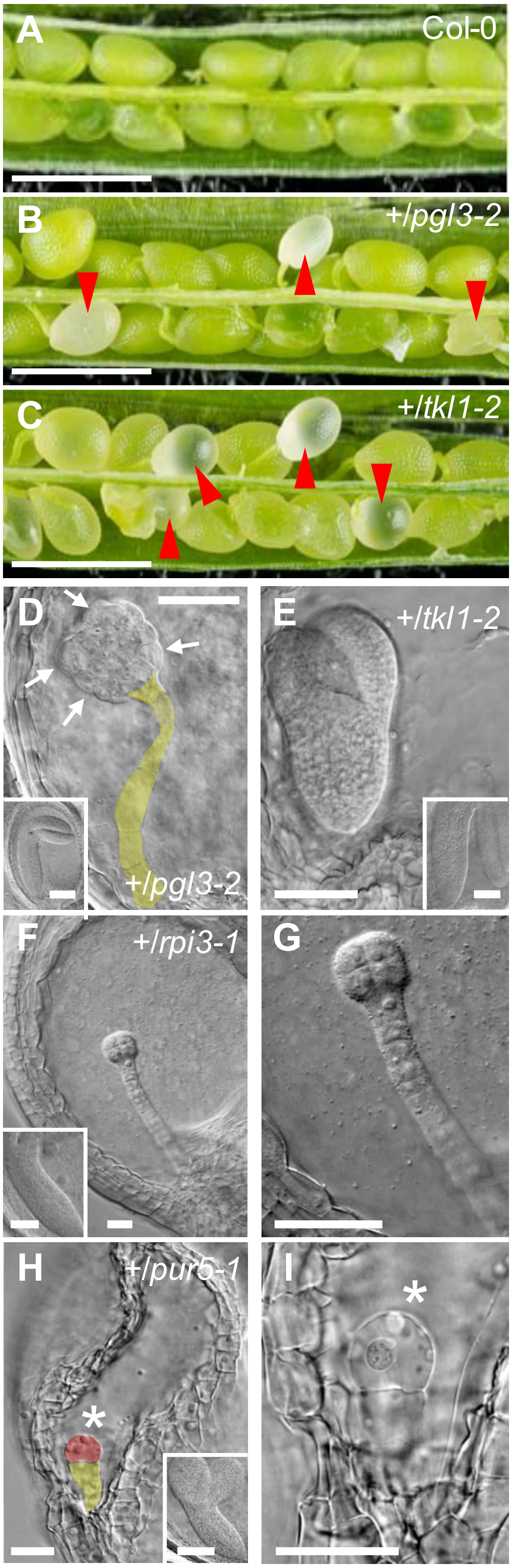

Fig. S2: Seed and embryo phenotypes in various mutants. Developing siliques from (*A*) wild-type, (*B*) +/*pgl3*-2, (*C*) +/*tkl1-2* plants. Arrowheads indicate white or aborting seeds. (*D*) Embryo from a white seed on a +/*pgl3*-2 plant. Arrows point to enlarged epidermal cells in the embryo proper. Inset, phenotypically normal sibling from the same silique. (*E*) Embryo from a white seed on a +/*tkl1-2* plant. Inset, phenotypically normal sibling from the same silique. (*F*) Embryo from an aborting seed on a +/*rpi3-1* plant. Inset, phenotypically normal sibling from the same silique. (*G*) Close-up view of the 8-cell embryo in *F*. (*H*) Aborting seed from a +/*pur5-1* plant with an embryo at the 1-cell stage. Inset: phenotypically normal sibling from the same silique. (*I*) Close-up view of the embryo in *H*. Asterisks in *H*, *I* point to the embryo proper. Seeds in *D-I* were imaged with DIC optics. In *D* and *H* false color has been added to highlight the embryo proper (red) and the suspensor (yellow). Scale bars: (*A-C*) 1 mm, (*D-I*) 20 µm.

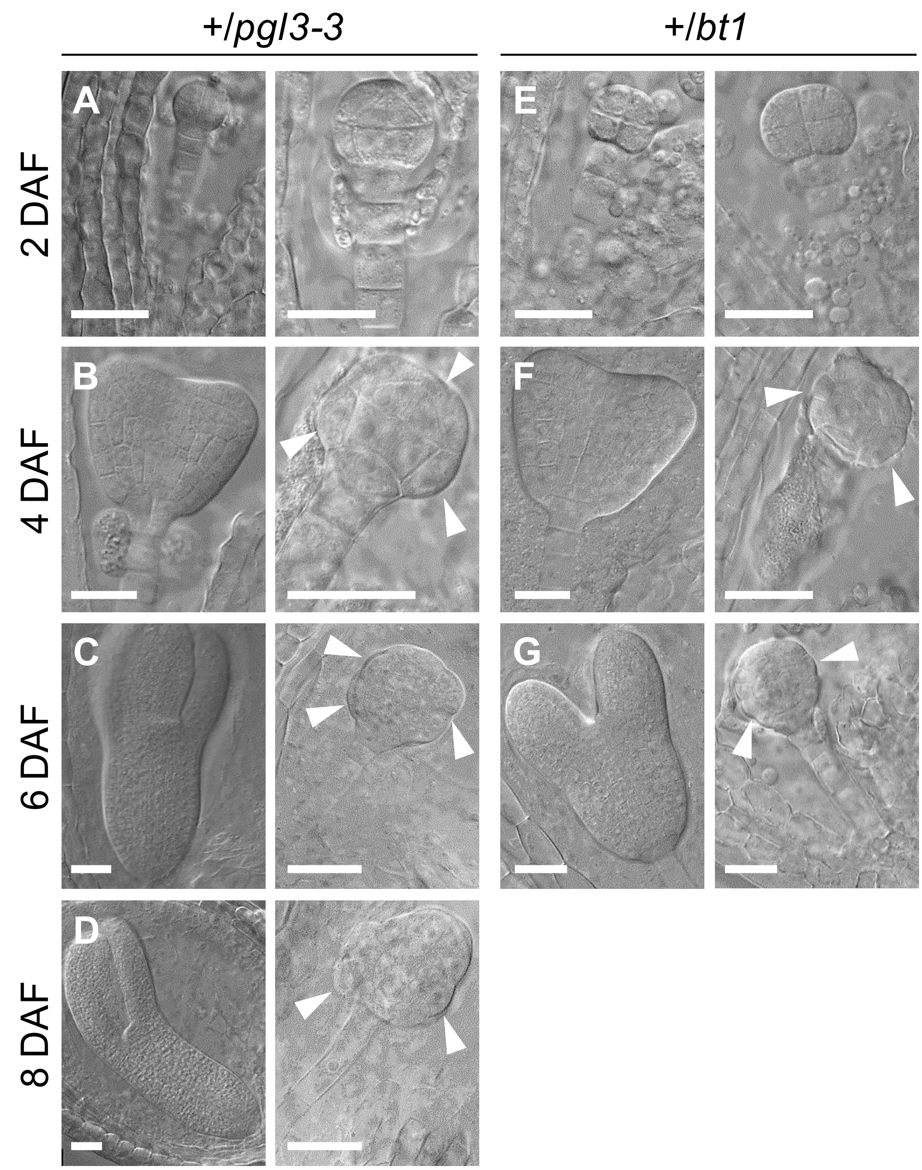

Fig. S3: Stages of developmental arrest in mutant embryos from +/*pgl3-3* and +/*bt-1* plants. Days after flowering (DAF) are indicated at right. All plants were grown at the same time in the same controlled environment growth room. (*A*-*D*) embryos from +/*pgl3-3* plants. At 2 DAF (*A*) all embryos were at the octant (left panel) to dermatogen (right panel) stage. In *B* to *D* phenotypically normal embryos from green seeds are pictured left, and embryos from white seeds are pictured right. Embryos from white seeds progressed no further than globular stage, and became raspberry-like as embryos from green seeds progressed through globular (*B*), torpedo (*C*) and walking-stick stage (*D*). (*E*-*G*) embryos from +/*bt1* plants. At 2 DAF (*E*) all embryos were at the octant (left panel) to dermatogen (right panel) stage. In *F* and *G* phenotypically normal embryos from green seeds are pictured left, and embryos from white seeds are pictured right. Embryos from white seeds progressed no further than globular stage, and became raspberry-like as embryos from green seeds progressed through heart (*F*) and torpedo stage (*G*). Embryos were viewed under DIC. Arrowheads point to enlarged, bulging protodermal cells in the embryo proper. Scale bars: 20 μm.

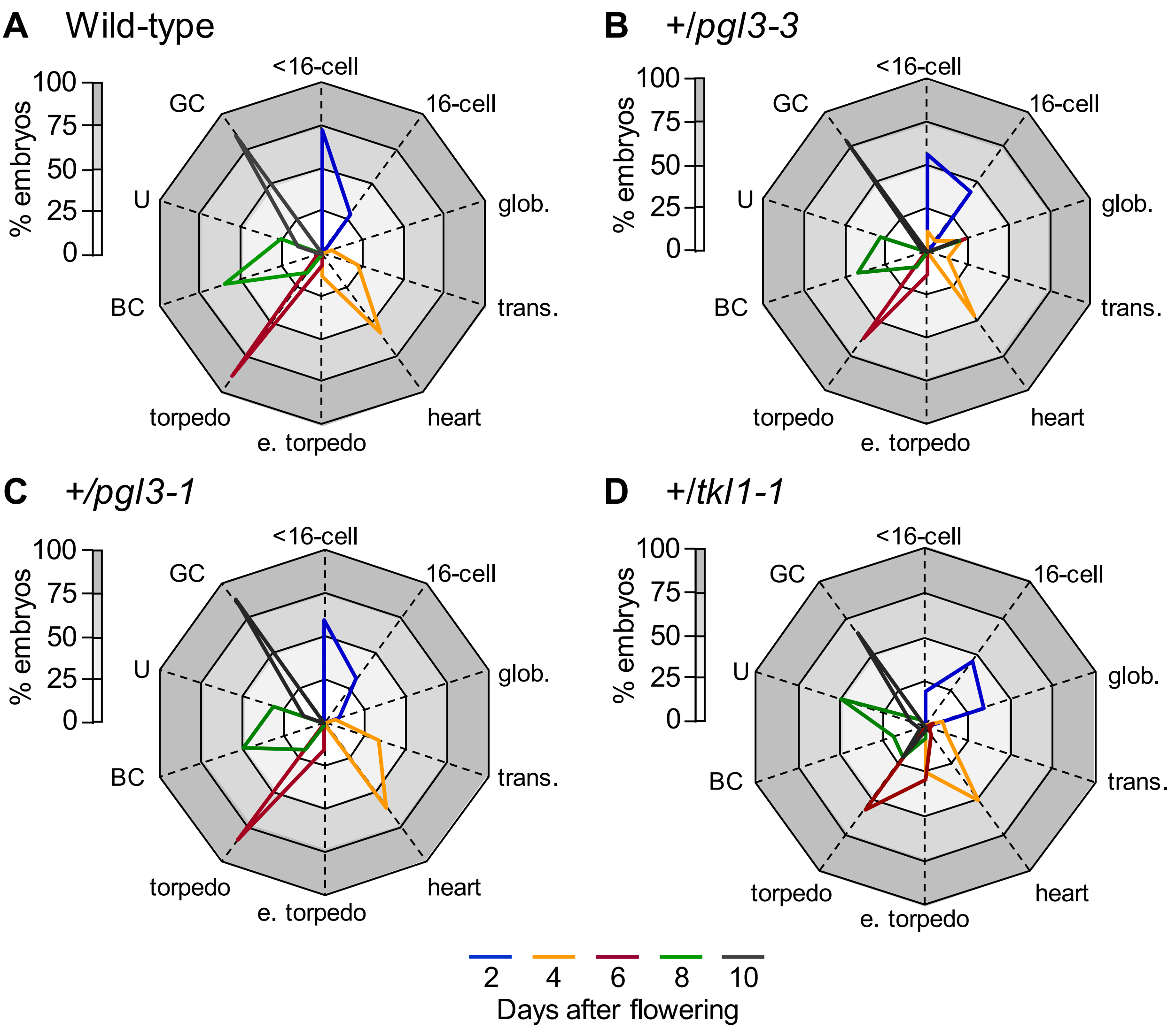

Fig. S4: Developmental progression of embryos from wild-type and heterozygous plants. Original data are in Table S4. Radar graphs show the developmental stages (at the periphery of the graph) of embryos at particular time points (indicated by the colour of the line, key at the base of the figure). Within each graph the white zone represents 0-25% of embryos examined, the light grey zone 25-50% of embryos, the grey zone 50-75% of embryos and the dark grey zone 75-100% of embryos. Note that *A* is the same as Fig. 4*A* and is shown to facilitate comparison with mutants in *B-D*. (*A*) wild-type, (*B*) +/*pgl3-3*, (*C*) *pgl3-1*, (*D*) +/*tkl1-1*.

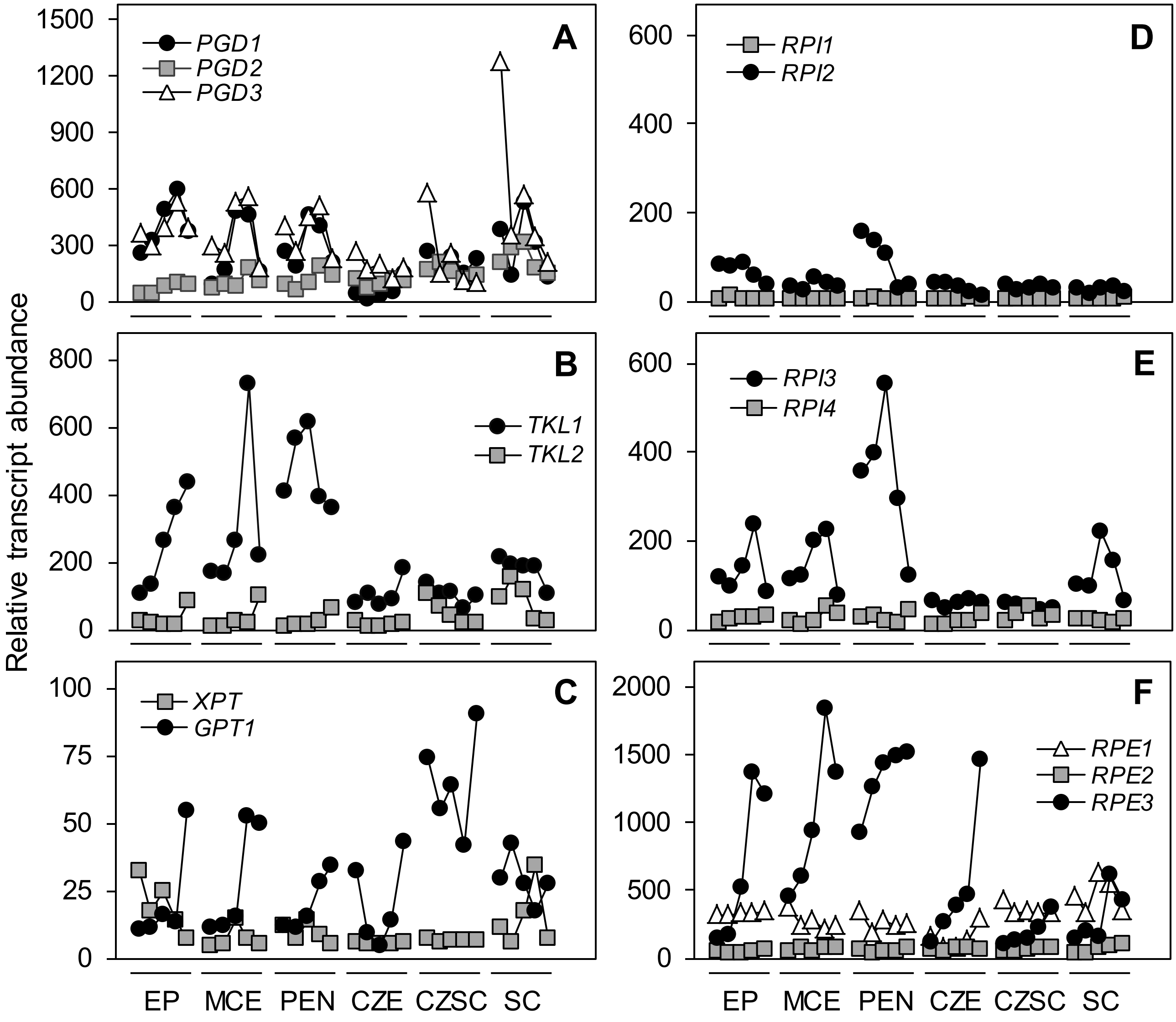

Fig. S5: Transcript abundance of genes encoding enzymes of the OPPP and of plastid envelope transporters in developing Arabidopsis seeds. (*A*) *PGD1*, *PGD2* and *PGD3* transcript, (*B*) *TKL1* and *TKL2* transcript, (*C*) *GPT1* and *XPT* transcript, (*D*) *RPI1* and *RPI2* transcripts (encoding cytosolic RPIs; 4), (*E*) *RPI3* and *RPI4* transcripts (encoding plastidial RPI enzymes; 4), (*F*) *RPE1*, *RPE2* and *RPE3* transcripts [encoding cytosolic (RPE1 and RPE2) and plastidial (RPE3) RPE enzymes; 4]. Results are from publicly-available transcriptomic data (http://seedgenenetwork.net/Arabidopsis; 5). In each data series the developmental stages are (from left to right): pre-globular, globular, heart, linear cotyledon (torpedo), green cotyledon stages. EP, embryo proper, MCE, micropylar endosperm; PEN, peripheral endosperm (syncytium); CZE, chalazal endosperm; CZSC, chalazal seed coat; SC, seed coat.

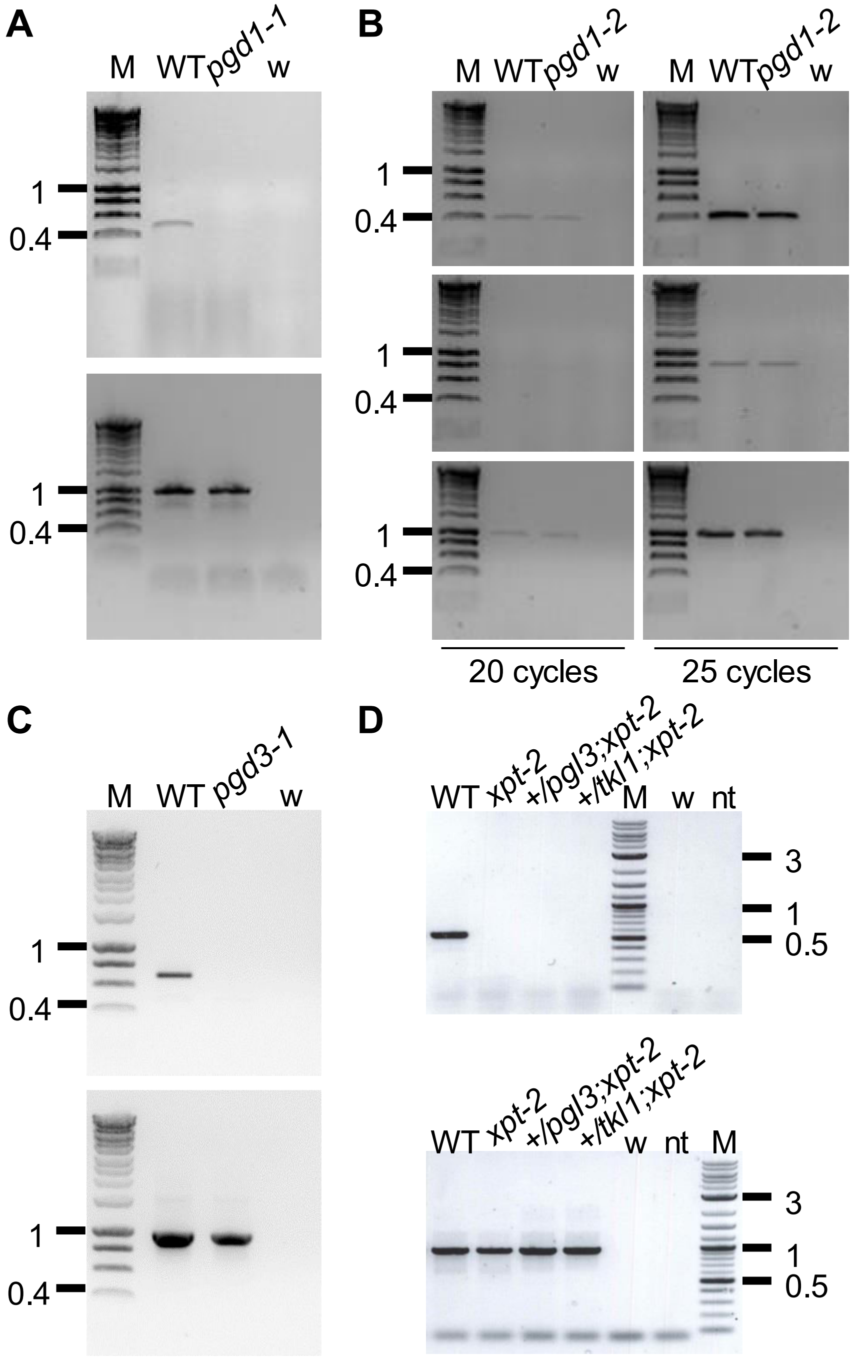

Fig. S6: Reverse transcriptase (RT) PCR analysis of gene expression in different mutant backgrounds. (*A*) Presence of *PGD1* transcript in wild-type (WT) and *pgd1-1* plants. cDNA was prepared from 2-weeks old seedlings. Top panel: *PGD1* transcript. Bottom panel: *TUB2* transcript. (*B*) Presence of *PGD1* transcript in wild-type (WT) and *pgd1-2* plants. cDNA was prepared from 2-weeks old seedlings. Amplification of either *PGD1* (top and middle rows) or *TUB2* (bottom row) transcripts was performed with different number of cycles (indicated at the bottom) during PCR. *PGD1* transcripts were amplified with primers VA734 and VA735 (top row) or VA148 and VA721 (middle row). (*C*) Presence of *PGD3* transcript in wild-type (WT) and *pgd3-1* plants. cDNA was prepared from 2-weeks old seedlings. Top panel: *PGD3* transcript. Bottom panel: *TUB2* transcript. (*D*) Presence of *XPT* transcript in wild-type (WT), *xpt-2*, *+/pgl3;xpt-2* and *+/tkl1;xpt-2* plants. cDNA was prepared from leaf tissue of 5-week old plants. RT-PCR was used to detect *XPT* transcript (top panel) and *TUB2* transcript (bottom panel). *TUB2* was used as a loading control. M is DNA markers of either the HyperLadder I (in *A*-*C*) or the 2-log DNA ladder (in *D*); sizes of major bands in kb are shown; nt, no-template control; w, negative control reaction in which water was substituted for cDNA. Gels within each panel were run and photographed at the same time.

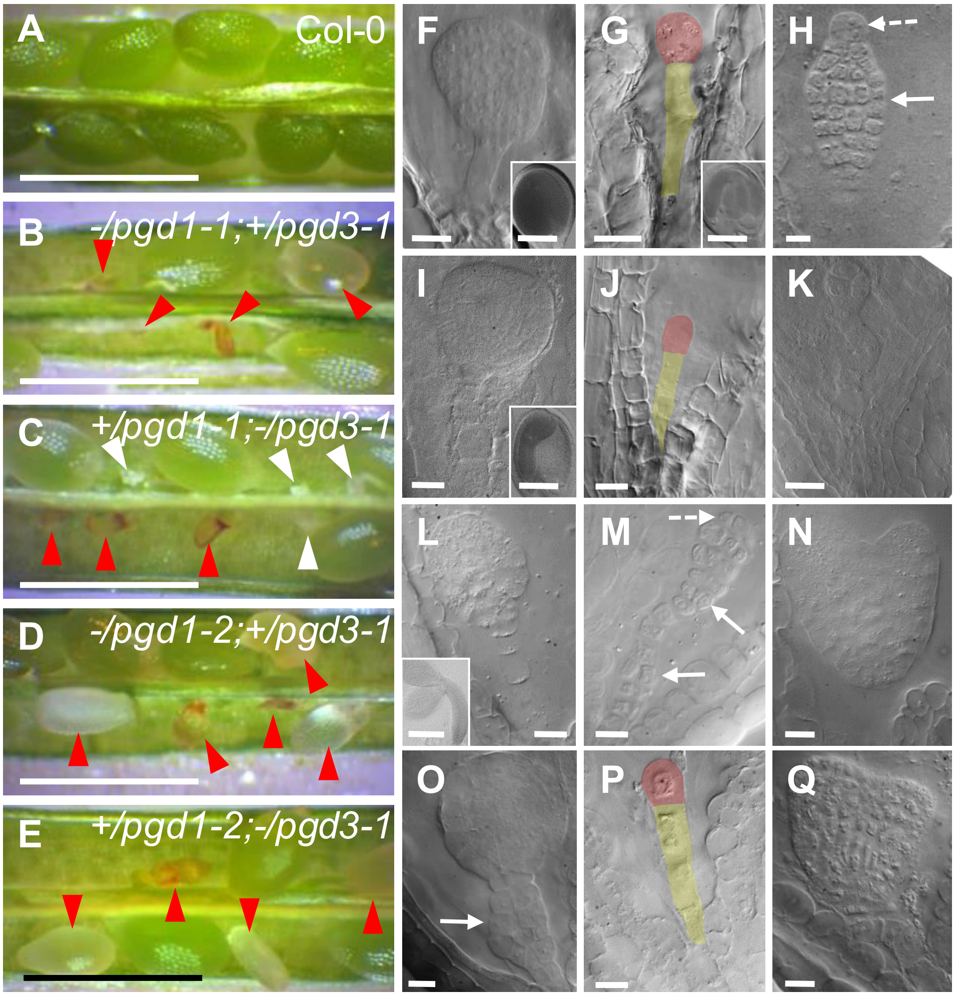

Fig. S7: Seed and embryo phenotypes in mutant plants lacking plastidial PGDs. Developing siliques from (*A*) wild-type, (*B*) *-/pgd1-1;+/pgd3-1*, (*C*) *+/pgd1-1;-/pgd3-1*, (*D*) *-/pgd1-2;+/pgd3-1*, (*E*) *+/pgd1-2;-/pgd3-1* plants. (*F-H*) Aborting embryos from siliques of *-/pgd1-1;+/pgd3-1* plants. (*F*) Embryo aborting at the globular stage having a raspberry-like appearance. Inset: Embryo from a green seed from the same silique, with an embryo at the green cotyledon stage. (*G*) Embryo aborting at the 2-cell stage. Inset: Embryo from a green seed from the same silique, with an embryo at the green cotyledon stage. (*H*) Embryo aborting at the pre-globular stage. The suspensor (arrow) is enlarged due to disorganised, transverse divisions giving rise to more than one cell file. The first division of the apical daughter cell in the embryo proper was longitudinal but the second division was transverse (dashed arrow) instead of longitudinal. (*I-K*) Aborting embryos from siliques of *+/pgd1-1;-/pgd3-1* plants. (*I*) Raspberry-like embryo. Inset: Inset: Embryo from a green seed from the same silique, with an embryo at the green cotyledon stage. (*J*) Embryo from the same silique as in *I*, aborting at the 1-cell stage. (*K*) Embryo aborted at the 8-cell stage. (*L-N*) Aborting embryos from siliques of *-/pgd1-2;+/pgd3-1* plants. (*L*) Raspberry-like embryo from a silique with embryos in green seeds at the green cotyledon stage (inset). (*M*) Aborting embryo in which the suspensor shows abnormal division planes (arrow) and the embryo proper stopped dividing after the first division that was longitudinal (dashed arrow). (*N*) Aborting embryo that reached the late heart-early torpedo stage of development. (*O-Q*) Aborting embryos from siliques of *+/pgd1-2;-/pgd3-1* plants. (*O*) Raspberry-like embryo with abnormal suspensor (arrow). Embryos in green seeds were at the green cotyledon stage. (*P*) Embryo aborting at the 1-cell stage. (*Q*) Aborting embryo that reached the heart stage of development. Scale bars: (*A-E*): 1mm, (*F-Q*): 10µm, insets in *F*,*G*,*I*,*L*: 20 µm. Red or open arrowheads in (*A-E*) point to abnormal seeds (white or small/collapsed seeds), or aborted/unfertilised ovules respectively. False colour has been added to highlight the embryo proper (red) and the suspensor (yellow) in *G*,*J*, and *P*.

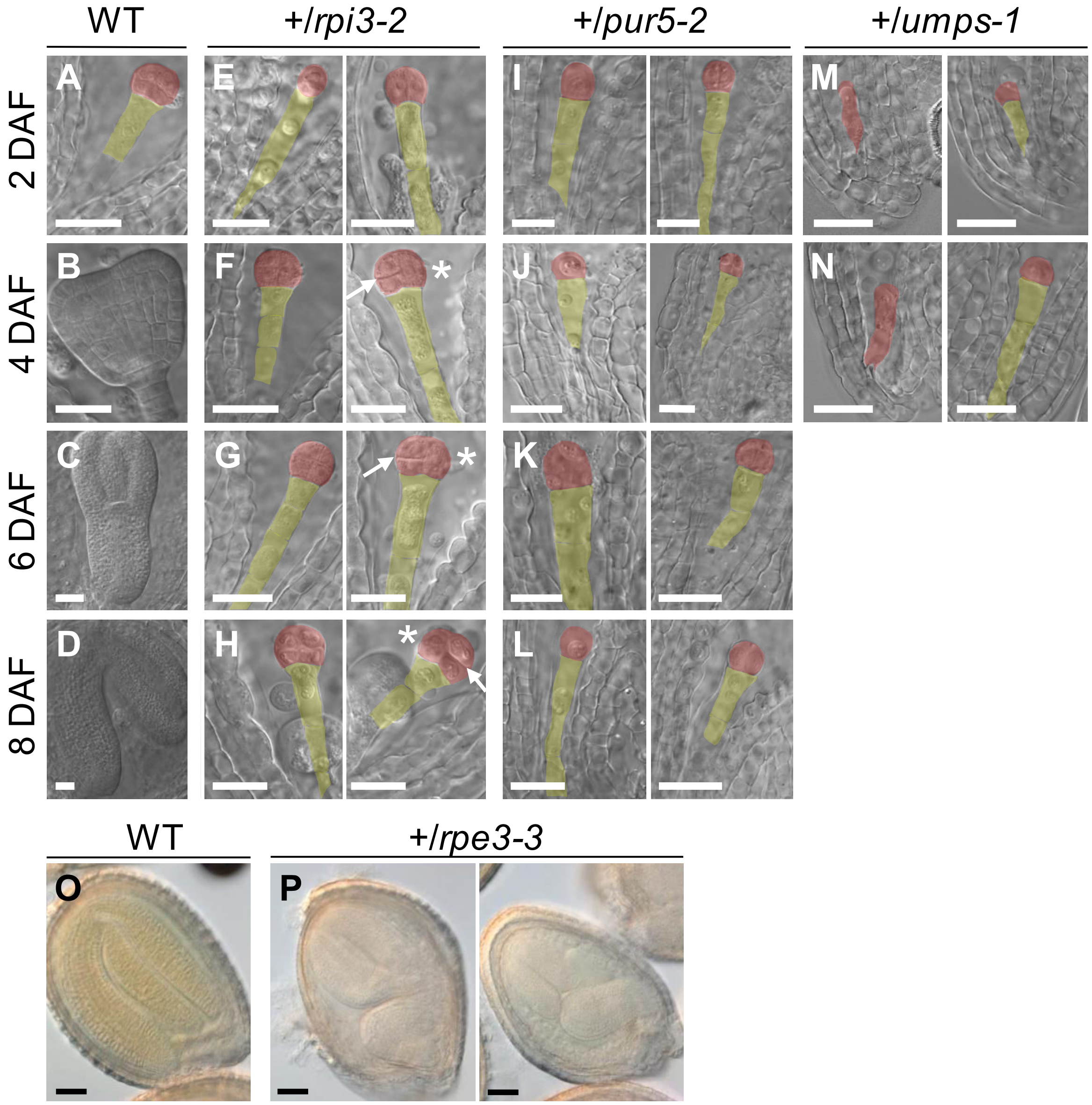

Fig. S8: Stages of developmental arrest in mutant embryos. (*A-N*) Embryos from wild-type, +/*rpi3-2*, +/*pur5-2* and +/*umps-1* plants. All plants were grown at the same time in the same conditions. Days after flowering (DAF) are indicated at right. Embryos were viewed under DIC optics. False colour has been added to highlight the embryo proper (red) and the suspensor (yellow). (*A-D*) Embryos from wild-type plants at the octant (*A*), heart (*B*), torpedo (*C*), walking-stick/upturned U (*D*) stages of development. Embryos from green seeds of heterozygous plants showed exactly the same developmental progression. (*E-H*) Embryo developmental progression at the same DAF in seeds destined for abortion on +/*rpi3-2* plants. At 2 DAF embryos were at the 1-cell (left panel) or 2-cell (right panel) stage (*E*). At 4 DAF (*F*) embryos were at the octant stage, and did not progress further (*G*, *H*: two examples at each stage). Asterisks show correctly performed first longitudinal division after the 1-cell stage; arrows show transverse instead of longitudinal second divisions. (*I-L*) As for *E-H* but for +/*pur5-2* plants. At 2 DAF embryos were at the 1-cell (left panel) or 2- to 4-cell (right panel) stage (I), and did not progress further (J-L: two examples at each stage). (M-N) As for E-H but for +/*umps-1* plants. At 2 DAF (M) nearly 60% of the embryos were at the zygote (left panel) and 1-cell (right panel) stages (also see Fig. 4*D*). At 4DAF (*N*) embryos were at the elongated zygote (left panel) or 1-cell (right panel) and did not progress any further. (*O*, P) Seeds from a +/*rpe3-3* plant viewed under DIC optics. (*O*) Phenotypically normal seed with an embryo at the green cotyledon stage of development. (*P*) Two pale-green seeds from the same silique as (*O*) with embryos that fill the seeds but lack the characteristic U-shape of wild-type embryos. Scale bars: (*A-N*): 20μm; (*O*,*P*): 50μm.

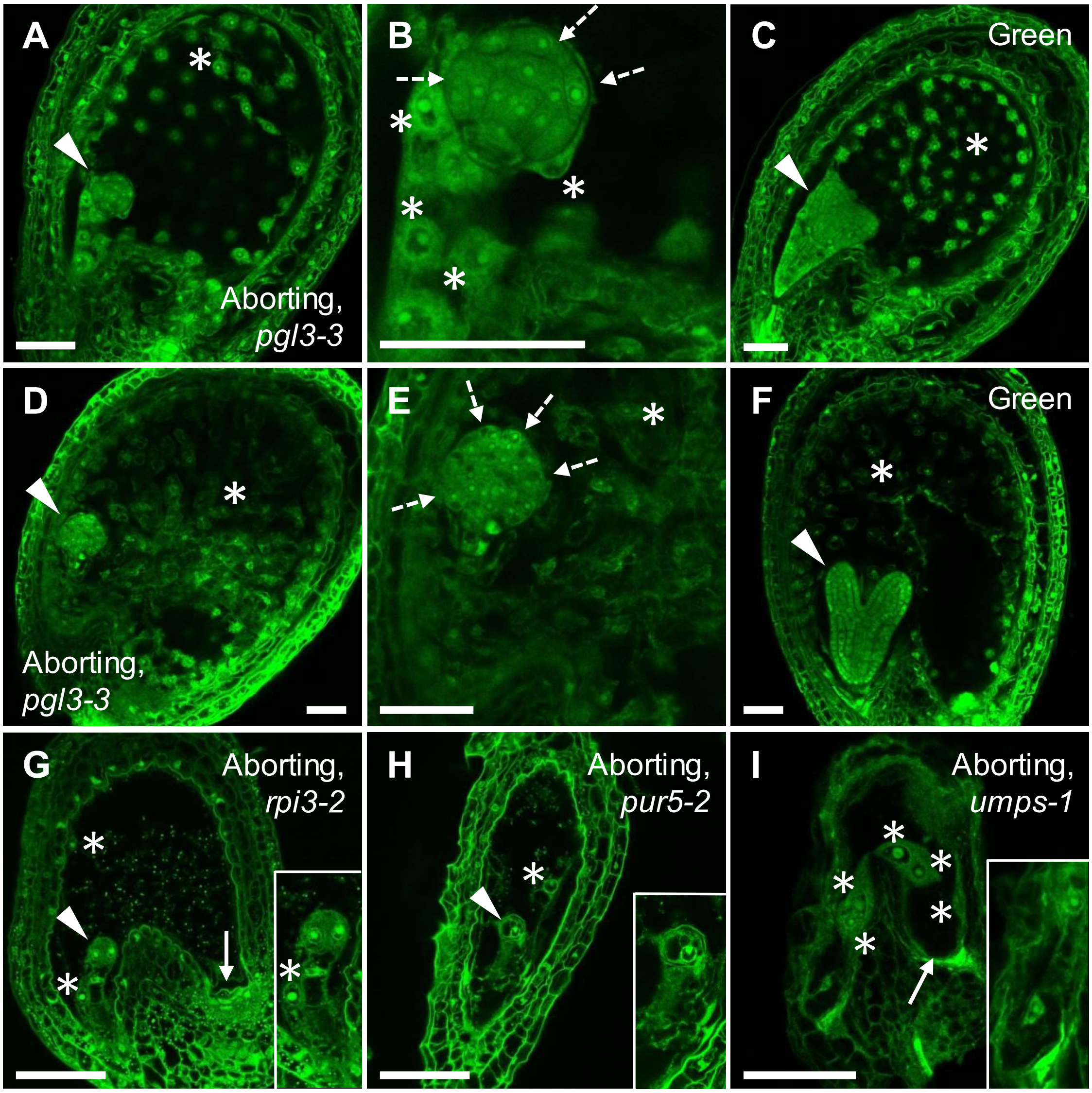

Fig. S9: Endosperm development is normal in white seeds from siliques on +/*pgl3-3* plants. (*A*) White seed from a +/*pgl3-3* plant with embryo transitioning to a raspberry-like appearance. (*B*) Close-up of embryo in *A*. (*C*) Green seed from the same silique as in *A*, with phenotypically normal embryo at the heart stage. Note that development of the syncytial endosperm is similar in *A* and *C*. (*D*) White seed from +/*pgl3-3* plant with a raspberry-like embryo. (*E*) Close-up of embryo in *D*. (*F*) Green seed from the same silique as *D*, with phenotypically normal embryo at the torpedo stage. Note that the endosperm is fully-developed and cellularized in both *F* and *D*. (*G*) Aborting seed from a +/*rpi3-2* plant with embryo arrested at the 4-cell stage. (*H*) Aborting seed from a +/*pur5-2* plant with embryo arrested at the 1- or 2-cell stage. (*I*) Aborting seed from a +/*umps-1* plant with embryo arrested at the zygote-1-cell stage. Insets in *G-I*: close-up of the micropylar end of the seed. Seeds were Feulgen-stained and imaged with a confocal microscope. Arrowheads indicate the embryo proper; dashed arrows indicate abnormally enlarged and bulging cells at the periphery of aborting *pgl3* embryos; arrow in *G*, *I* point to the chalazal-end of the seed. The chalazal cyst failed to develop; asterisks indicate endosperm nuclei (in *A*-*C*, *G*-*I)* or cellularized endosperm (in *D*-*F*). Scale bars: (*A*-*F*) 40 µm, (*G*-*I*) 50 µm.

Table S1. Segregation ratios of arrested to normal embryos in siliques on selfed heterozygous or homozygous mutant plants.

Parental genotype	Seeds examined (total)	Seed classes		Chi-square[a]
		Normal	Abnormal	
+/*pgl3-2*	416	317 (76.2%)	99 (23.8%)	0.57
+/*pgl3-3*	216	166 (77%)	50 (23%)	0.53
pgd1-1	285	285	0	10^{-22}x2.4 [b]
pgd1-2	261	260 (99.6%)	1 (0.4%)	10^{-20}x4.1
pgd3-1	327	320 (97.9%)	7 (2.1%)	10^{-21}x1.3
+/*rpi3-2*	89	65 (73.1%)	24 (26.9%)	0.67
+/*tkl1-1*	415	304 (73.2%)	111 (26.8%)	0.41
+/*tkl1-2*	628	464 (73.8%)	164 (26.2%)	0.52
xpt-2	295	295	0	10^{-23}x3.5
+/*pgl3-3*;*xpt-2*	364	268 (73.6%)	96 (26.3%)	0.54
+/*tkl2-1*;*xpt-2*	385	299 (77.6%)	86 (22.3%)	0.22
+/*pur5-1*	906	724 (79.9%)	182 (20.1%)	0.0006
+/*pur5-2*	777	605 (78%)	172 (22%)	0.065
+/*umps-1*	205	168 (82%)	37 (18%)	0.021
+/*cs-1*	221	164 (74%)	57 (26%)	0.79
+/*rpe3-2*	362	266 (73.5%)	96 (26.5%)	0.50
+/*rpe3-3*	632	460 (72.8%)	172 (27.2%)	0.20

Parental genotype	Seeds examined (total)	Phenotypic classes		
		Normal	Abnormal	Ovules
-/*pgd1-1*;+/*pgd3-1*	653	389	128	136
+/*pgd1-1*;-/*pgd3-1*	333	196	77	60
-/*pgd1-2*;+/*pgd3-1*	845	496	295	54
+/*pgd1-2*;-/*pgd3-1*	2149	1118	826	205
Col-0 wild-type	192	188	0	4

[a]Probability that there is a 1:3 ratio of abnormal to normal seeds.

[b]Chi square values of <0.05 (indicating deviation from 1:3 segregation) are in red.

Table S2. Segregation of mutant alleles in the progeny of heterozygous parental lines.

Gene	Parental genotype	Plants genotyped (total)	Genotype of progeny[a]			Chi-square
			+/+	+/-	-/-	
PGD1	*+/pgd1-1*	24	8	13	3	0.32 [b]
	+/pgd1-2	16	5	4	6	0.19
PGD3	*+/pgd3-1*	24	4	13	7	0.63
XPT	*+/xpt-2*	20	4	9	7	0.58
			+/+	+/-	-/-	
PGL3	*+/pgl3-3*	150	53	97	0	0.60[c]
RPI3	*+/rpi3-1*	20	7	13	0	0.88
	+/rpi3-2	67	21	46	0	0.73
TKL1	*+/tkl1-1*	32	9	23	0	0.53
	+/tkl1-2	35	10	25	0	0.55
PUR5	*+/pur5-1*	61	29	32	0	0.0185[d]
	+/pur5-2	44	23	21	0	0.0077
UMPS	*+/umps-1*	39	25	14	0	0.0000485
BT1	*+/bt-1*	22	8	14	0	0.75

[a]The genotype of the progeny of heterozygous parental lines was determined by PCR with gene-specific and T-DNA-specific primers (Table S5).

[b]Probability that there is a 1:2:1 ratio of wild type, heterozygous and homozygous mutant progeny.

[c]Probability that there is a 1:2 ratio of wild type to heterozygous progeny.

[d]Chi square values of <0.05 (indicating deviation from 1:2 segregation) are in red.

Table S3. Segregation of *pgd1* and *pgd3* mutant alleles in the progeny of selfed *+/pgd1;+/pgd3* parental lines.

Genotype	Expected	Observed[a]	
		+/pgd1-1;+/pgd3-1	*+/pgd1-2;+/pgd3-1*
+/PGD1;+/PGD3	3	5	1
+/PGD1;+/pgd3	6	9	9
+/PGD1;-/pgd3	3	3	5
+/pgd1;+/PGD3	6	7	9
+/pgd1;+/pgd3	12	15	14
+/pgd1;-/pgd3	6	1	4
-/pgd1;+/PGD3	3	3	4
-/pgd1;+/pgd3	6	5	2
-/pgd1;-/pgd3	3	0	0
Total	48	48	48

[a]The genotype of the progeny of selfed *+/pgd1-1;+/pgd3-1* or *+/pgd1-2;+/pgd3-1* parental lines was determined by PCR with primers (Table S5) specific for the wild-type *PGD1* or *PGD3* alleles and with primers specific for the T-DNA mutant allele in *pgd1-1* (GABI_762C02), *pgd1-2* (SALK_121521) and *pgd3-1* (SAIL_528_E08).

Table S4. Phenotypes of embryos from siliques growing on Wild-type, *pgl3-1*, *+/pgl3-3*, *+/tkl1-2*, *+/rpi3-2*, *+/pur5-2*, *+/bt-1* and *+/umps-1* Arabidopsis plants through seed development.

Wild-type (Col-0)

	pre-globular												
DAF	1-cell	2/4-cell	8-cell	derm	glob	trans	heart	early torp	torp	BC	EC	GC	total
2	-	25	63	35	-	-	-	-	-	-	-	-	123
4	-	-	-	-	8	33	84	20	-	-	-	-	145
6	-	-	-	-	-	-	-	11	144	6	-	-	161
8	-	-	-	-	-	-	-	-	24	99	42	-	165
10	-	-	-	-	-	-	-	-	-	1	22	131	154

pgl3-1

	pre-globular												
DAF	1-cell	2/4-cell	8-cell	derm	glob	trans	heart	early torp	torp	BC	EC	GC	total
2	-	19	47	35	10	-	-	-	-	-	-	-	111
4	-	-	-	-	8	40	74	-	-	-	-	-	122
6	-	-	-	-	-	-	-	32	172	-	-	-	204
8	-	-	-	-	-	-	-	3	33	86	54	-	176
10	-	-	-	-	-	-	-	-	-	-	17	122	139

+/pgl3-3

	pre-globular												
DAF	1-cell	2/4-cell	8-cell	derm	glob	trans	heart	early torp	torp	BC	EC	GC	total
2	25	34	61	90	2	-	-	-	-	-	-	-	212
4	1	18	18	25	65	42	149	-	-	-	-	-	318
6	-	-	-	-	69	-	-	42	188	2	-	-	301
8	-	-	-	-	47	-	-	-	26	101	68	-	242
10	-	-	-	-	61	-	-	-	1	-	10	262	334

+/tkl1-2

	pre-globular												
DAF	1-cell	2/4-cell	8-cell	derm	glob	trans	heart	early torp	torp	BC	EC	GC	total
2	2	4	19	58	44	-	-	-	-	-	-	-	127
4	-	-	-	1	25	29	122	62	-	-	-	-	239
6	-	-	-	-	13	7	16	81	158	-	-	-	275
8	-	-	-	-	-	-	-	15	50	44	119	11	239
10	-	-	-	-	-	-	-	4	39	6	15	119	183

+/rpi3-2

	pre-globular												
DAF	1-cell	2/4-cell	8-cell	derm	glob	trans	heart	early torp	torp	BC	EC	GC	total
2	30	45	52	59	6	-	-	-	-	-	-	-	192
4	18	17	37	5	31	45	115	9	-	-	-	-	277
6	-	18	44	5	-	-	-	63	131	-	-	-	261
8	-	3	21	-	-	-	-	-	9	23	33	-	89

+/pur5-2

DAF	pre-globular 1-cell	2/4-cell	8-cell	derm	glob	trans	heart	early torp	torp	BC	EC	GC	total
2	44	21	33	67	22	-	-	-	-	-	-	-	187
4	21	23	-	2	51	48	75	-	-	-	-	-	220

+/bt1

DAF	pre-globular 1-cell	2/4-cell	8-cell	derm	glob	trans	heart	early torp	torp	BC	EC	GC	total
2	2	21	22	59	3	-	-	-	-	-	-	-	107
4	-	-	-	7	36	29	41	-	-	-	-	-	113
6	-	-	-	-	49	-	-	41	140	-	-	-	230

+/umps-1

DAF	zygote	pre-globular 1-cell	2/4-cell	8-cell	derm	glob	trans	heart	early torp	torp	BC	EC	GC	total
2	17	27	11	45	61	7	-	-	-	-	-	-	-	168
4	6	30	-	-	4	33	38	70	11	-	-	-	-	192

Phenotypic classes are: derm, dermatogen; glob, globular; torp, torpedo; BC, bent cotyledon (walking stick); EC, expanded cotyledon (upturned-U); GC, green cotyledon. Numbers are the embryos in each phenotypic class observed under DIC at the indicated days after flowering (DAF).

Table S5. Oligonucleotides used in this study.

Genotyping of plants carrying T-DNA alleles				
Primer	Gene	Mutant allele	T-DNA mutant line	Sequence[a]
VA15	At5g24400	*pgl3-2*	FLAG_219G10	ACCAGAACGGAACCAAAGATC
VA16				AGGCGGAATTGGTACCTAAAG
VA7		*pgl3-3*	emb2024	CATCGATCGGAACTGGATCT
VA8				CACGGAGAATGTTGGTGTTG
VA200	At3g04790	*rpi3-1*	RATM11-0136-1H	CCAATGGCTTCCTTATCCTTC
VA201				CAATCCGACTCAACTTTTTGC
VA110		*rpi3-2*	emb3119-2	ACTTCCCAATCTCTTTAGCCG
VA111				AATCTTCCCACCCAAGAATTG
VA102	At5g17630	*xpt-2*	SAIL_378_C01	AAAAGACAAATGATGGCATCG
VA103				TAACGGATACGAATCACCGAG
VA134		*xpt-4*	GT_5_112515	TCAGCTAGGGATTGTGTTTGG
VA135				GGAAAAACATCAAGTAGACGAGG
VA190	At3g60750	*tkl1-1*	WiscDsLox453_456I14	GGTGAAAGTCTTTGCGTCTTG
VA191				TGCCCAACACTCTTATGTTCC
VA271		*tkl1-2*	SAIL_58_D02	TCCAACGGTCATATTTGATCC
VA272				GATTCCTTGTCCAAGAGGACC
VA196	At3g55010	*pur5-1*	SALK_070673	ATGTTGTCTGTGAAACCTCCG
VA197				TTAAACGAATCGCAAAGATGG
VA198		*pur5-2*	SAIL_343_A07	TGTTTGTTTGTGGTATGCTGC
VA199				AGGTGGATCAAGTACCCTTGG
VA279	At3g54470	*umps-1*	FLAG_038G05	TTGTGGGCTTGCAATTTTATC
VA280				TATGATTGCAGGGTATGCCTC
VA194	At1g64190	*pgd1-1*	GABI_762C06	TATGGGTGGTTCTTGTCAAGG
VA195				ACCGTTACGAGCTCCTTCTTC
VA148		*pgd1-2*	SALK_121521	CTGAGTGGAATTCAGGTGAGC
VA149				TTTTGCTAATCGGATGTTTGG
VA716	At5g41670	*pgd3-1*	SAIL_528_E08	TGCGAGTAGAAAAGATACGCC
VA717				GACCATCTTCGACTTGAGCAG
VA712		*pgd3-2*	SALK_040050	GGACAAAACCTCGCCTTAAAC
VA713				AAACCGCCCTGATAATACACC
VA714		*pgd3-3*	SALK_202519	ATTCGAAAAATGAACCAAGCC
VA715				GAATTGGCTGAGATCTTCACG

T-DNA specific oligonucleotides

Primer	T-DNA lines	Sequence[a]
LBa1	SALK T-DNA lines	TGGTTCACGTAGTGGGCCATCG
LBb1.3	SALK T-DNA lines	ATTTTGCCGATTTCGGAAC
RB1	SALK T-DNA lines	AGCGTTCGAGCAGGGACTC
Sail_LB1	SAIL T-DNA lines	GCCTTTTCAGAAATGGATAAATAGCCTTGCTTCC
Ds3-1	GT lines	ACCCGACCGGATCGTATCGGT
Ds5-2a	RIKEN RATM lines	TCCGTTCCGTTTTCGTTTTTTAC
Ds3-2a	RIKEN RATM lines	CCGGATCGTATCGGTTTTCG
F-LB1	FLAG lines	CGGCTATTGGTAATAGGACACTGG
F-LB4	FLAG lines	CGTGTGCCAGGTGCCCACGGAATAGT
p745	WiscDsLOX lines	AACGTCCGCAATGTGTTATTAAGTTGTC

Sequencing

Primer	Sequence[a]
M13FW	GTAAAACGACGGCCAG
M13RV	CAGGAAACAGCTATGAC

RT-PCR

Primer	*Gene*	Sequence[a]
VA291	*XPT* (At5g17630)	AACGGATCTCTGACGTTTGG
VA293		AGCTGAAACGAAGCGAGAAG
VA721	*PGD1* (At1g64190)	TCAATGGTTCTTCCTGGCAAG
VA732		GCTCATCTCTCTATCTCCAATGG
VA733		CCACCAGGCATTAACGAAGG
VA734		TGGCTAGGATTTGGAAAGGTG
VA735		AGCAAGACAAATAGGGCTTTTAC
VA148		CTGAGTGGAATTCAGGTGAGC
VA722	*PGD3* (At5g41670)	GAGTCCGTCGCTCTATCTCGC
VA743		AGCTCACCTCGATTCCACTC
VA287	*TUB2* (At5g62690)	GTGAACTCCATCTCGTCCAT
VA288		CCTGATAACTTCGTCTTTGG

[a]All primer sequences are in the 5'→3' orientation.

Supporting Videos

Movie S1. *z*-stack of the wild-type seed shown in Fig. 6*A*, with embryo at 2/4-cell stage. The endosperm and chalazal cyst are normal. The seed was Feulgen stained and imaged under a confocal microscope. Optical sections were acquired at 0.5-nm intervals covering the seed cavity. The movie was created from a z-stack of 40 optical sections. Scale bar is 40 µm.

Movie S2. *z*-stack of the wild-type seed shown in Fig. 6*B*, with embryo at octant stage. The endosperm and chalazal cyst are normal. The seed was Feulgen stained and imaged under a confocal microscope. Optical sections were acquired at 0.5-nm intervals covering the seed cavity. The movie was created from a z-stack of 28 optical sections. Scale bar is 40 µm.

Movie S3. *z*-stack of the aborting seed from a *+/rpi3-2* plant, shown in Fig. 6*E*, when embryos in green seeds from the same silique were at the torpedo stage. The embryo had arrested development at the 8-cell/dermatogen stage of development. The peripheral endosperm has fewer nuclei than in wild-type seeds of comparable developmental stage; the nuclei are unevenly distributed along the anterior-posterior axis. The chalazal cyst failed to develop. The seed was Feulgen stained and imaged under a confocal microscope. Optical sections were acquired at 0.5-nm intervals, covering the seed cavity. The movie was created from a z-stack of 19 optical sections. Scale bar is 15 µm.

Movie S4. *z*-stack of the aborting seed shown in Fig. 6*I* from a *+/pur5-2* plant, when embryos in green seeds from the same silique were at the heart stage. The embryo had arrested development at the 2/4-cell stage. The peripheral endosperm has fewer nuclei that are unevenly distributed along the anterior-posterior axis, compared to seeds of comparable developmental stage from wild-type plants. The chalazal cyst failed to develop. The seed was Feulgen stained and imaged under a confocal microscope. Optical sections were acquired at 0.5-nm intervals from the top of the seed coat. The movie was created from a z-stack of 96 optical sections. Scale bar is 35 µm.

Movie S5. *z*-stack of the aborting seed from a *+/umps-1* plant, shown in Fig. 6*M*, when embryos in green seeds from the same silique were at the late globular-transition stage. The embryo had arrested at the elongated zygote/1-cell stage. Endosperm development had ceased after the first mitotic divisions and the endosperm nuclei appear to be enlarged. The chalazal cyst failed to develop. The seed was Feulgen stained and imaged under a confocal microscope. Optical sections were acquired at 0.5-nm intervals from the top of the seed coat. The movie was created from a z-stack of 44 optical sections. Scale bar is 25 µm.

SI References

1. V.M.E. Andriotis, N.J. Kruger, M.J. Pike, A.M. Smith, Plastidial glycolysis in developing Arabidopsis embryos. *New Phytol.* **185**, 649-662 (2010).
2. V.M.E. Andriotis, M.J. Pike, B. Kular, S. Rawsthorne, A.M. Smith, Starch turnover in developing oilseed embryos. *New Phytol.* **187**, 791-804 (2010).
3. D.D. Figueiredo, R.A. Batista, P.J. Roszak, C. Köhler, Auxin production couples endosperm development to fertilization. *Nat. Plants* **23**, 15184 (2015).
4. N.J. Kruger, A. von Schaewen, The oxidative pentose phosphate pathway: structure and organisation. *Curr. Opin. Plant Biol.* **6**, 236–246 (2003).
5. M.F. Belmonte *et al.*, Comprehensive developmental profiles of gene activity in regions and subregions of the Arabidopsis seed. *Proc. Natl. Acad. Sci. U.S.A.* **110**, E435-E444 (2013).
